# A short-term, high-caloric diet has prolonged effects on brain insulin action in men

**DOI:** 10.1038/s42255-025-01226-9

**Published:** 2025-02-21

**Authors:** Stephanie Kullmann, Lore Wagner, Robert Hauffe, Anne Kühnel, Leontine Sandforth, Ralf Veit, Corinna Dannecker, Jürgen Machann, Andreas Fritsche, Nobert Stefan, Hubert Preissl, Nils B. Kroemer, Martin Heni, André Kleinridders, Andreas L. Birkenfeld

**Affiliations:** 1 Institute for Diabetes Research and Metabolic Diseases of the Helmholtz Center Munich at the University of Tübingen, Tübingen, Germany; 2 German Center for Diabetes Research (DZD), Neuherberg, Germany; 3 Department of Internal Medicine, Division of Diabetology, Endocrinology and Nephrology, Eberhard Karls University Tübingen, Tübingen, Germany; 4 Institute of Nutritional Science, Department of Molecular and Experimental Nutritional Medicine, University of Potsdam, Nuthetal, Germany; 5 German Institute of Human Nutrition, Junior Research Group Central Regulation of Metabolism, Nuthetal, Germany; 6 Section of Medical Psychology, Department of Psychiatry and Psychotherapy, Faculty of Medicine, University of Bonn, Bonn, Germany; 7 Section on Experimental Radiology, Department of Radiology, University Hospital Tübingen, Tübingen, Germany; 8 Department of Psychiatry and Psychotherapy, Tübingen Center for Mental Health (TüCMH), University of Tübingen, Tübingen, Germany; 9 Institute for Clinical Chemistry and Pathobiochemistry, Department for Diagnostic Laboratory Medicine, Eberhard Karls University Tübingen, Tübingen, Germany; 10 Division of Endocrinology and Diabetology, Department of Internal Medicine I, University of Ulm, Ulm, Germany

**Keywords:** Feeding behaviour, Metabolism, Functional magnetic resonance imaging, Endocrine system and metabolic diseases, Translational research

## Abstract

Brain insulin responsiveness is linked to long-term weight gain and unhealthy body fat distribution. Here we show that short-term overeating with calorie-rich sweet and fatty foods triggers liver fat accumulation and disrupted brain insulin action that outlasted the time-frame of its consumption in healthy weight men. Hence, brain response to insulin can adapt to short-term changes in diet before weight gain and may facilitate the development of obesity and associated diseases.

## Abstract (web summary)

Short-term overeating with calorie-rich snacks is shown to disrupt insulin action in the brain of men, which outlasted the time-frame of overeating.

## Main

Insulin resistance is a common feature of obesity and type 2 diabetes with detrimental effects in the periphery^1^ and the central nervous system^2^. In the healthy state, insulin acts in the brain in an anorexigenic fashion reducing appetite and food intake^3^, whereas in the insulin-resistant state, brain insulin action no longer properly regulates peripheral energy metabolism and feeding behaviour^2,4,5^. Concurrently, people with aberrant insulin response have higher visceral adipose tissue mass and impaired peripheral metabolism^6–8^ and regain more fat mass after a lifestyle intervention^7^. Furthermore, findings from numerous studies suggest that the disruption of insulin responsiveness in the human brain promotes metabolic, psychiatric and neurodegenerative diseases^4,9^. However, the developmental trajectory of brain insulin responsiveness in humans is currently unclear. To close this gap, we investigated the effect of a 5-day high-caloric diet (HCD) that included broadly available and commonly consumed, calorie-rich ultra-processed snacks in addition to the regular diet, compared with a regular diet on brain insulin action, body fat composition and peripheral insulin sensitivity. To study the brain-specific effects of insulin action, intranasal insulin (INI) application was used as a noninvasive method for the delivery of insulin to the brain in combination with functional magnetic resonance imaging (fMRI). Our primary aim was to assess brain insulin activity before, directly after the HCD compared with a normal caloric diet and 1 week after the return to a regular diet. Previous experimental findings indicate sex differences in the response to INI affecting appetite, metabolism and memory function^3,10^. Hence, we only evaluated brain insulin action in response to overeating in healthy weight male participants to investigate the temporal dynamics of brain insulin action in response to an unhealthy diet.

**Fig. 1:**
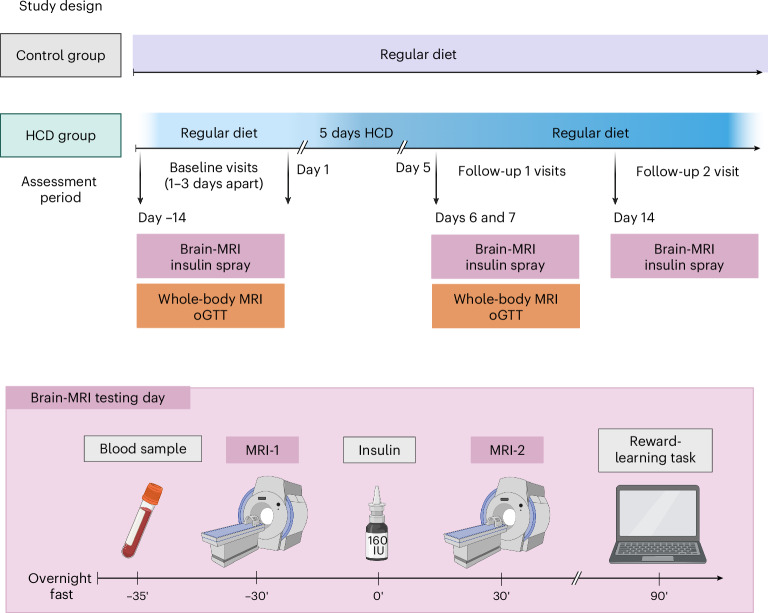
Schematic overview of the study design. After initial screening, healthy weight male participants underwent two baseline assessment days after an overnight fast at ~08:00. On the brain MRI testing day, diffusion-weighted imaging and CBF responses to 160 IU INI were acquired to investigate white matter integrity and brain insulin action (∆CBF = CBF MRI-2 − CBF MRI-1), respectively, followed by a reward-learning task. On a separate testing day (1–3 days apart), whole-body MRI for measurement of body fat mass and distribution and oGTTs for measurement of peripheral insulin sensitivity were performed. Afterwards, 18 participants were instructed to increase their daily caloric intake by 1,500 kcal for five consecutive days with high-caloric snacks. Eleven participants maintained their regular diet. Both testing days were repeated immediately after the 5-day HCD or regular diet period at follow-up 1. At follow-up 2, the brain MRI testing day was repeated 7 days after resuming a regular diet. Eating behaviour questionnaires were acquired during all testing days. Between visits, participants recorded their food intake and daily step activity. The timing of the follow-up visits was adapted to the first day of the HCD recording. Figure created in BioRender^50^.

**Extended Data Fig. 1:**
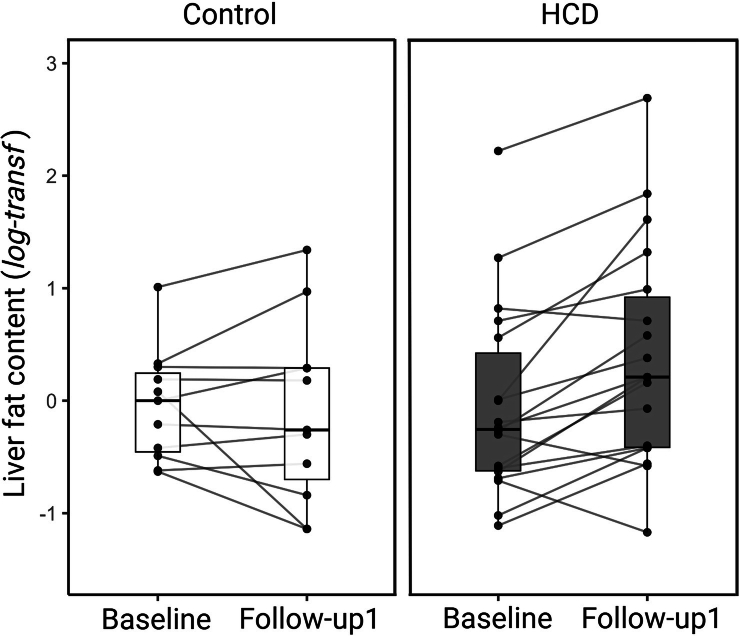
Liver fat content in the control and high-caloric diet group (HCD) at baseline and 5-days after the high-caloric or regular diet intervention (follow-up 1). Liver fat content increased in the HCD group: baseline versus follow-up 1: p = 0.005, while it did not change in the control group (p = 0.958). Presented are box plots with whiskers indicating 1.5 interquartile range and line diagram; Control: N = 11 at baseline and follow-up 1; HCD: N = 17 at baseline and N = 18 follow-up 1. Abbreviations: HCD, high-caloric diet group. Source data

In a nonrandomized controlled design, a total of 29 male volunteers (age 19–27 years, body mass index (BMI) 19–25 kg m^−2^) enrolled to participate either in a 5-day HCD (*n* = 18) or a regular diet (*n* = 11; no additional calories) (Table 1). Participants completed three visits (baseline, follow-up 1 and follow-up 2) during an assessment period of approximately 3–4 weeks (see Fig. 1 for study design). Seventeen of the HCD group completed all three visits. The HCD group were instructed to increase their daily caloric intake by 1,500 kcal for five consecutive days with high-caloric ultra-processed snacks 5 days before follow-up 1 visit. Thereafter, HCD group participants resumed their regular diet for 7 days before follow-up 2. Eleven participants maintained their habitual diet throughout the study. The food diary showed that the HCD group increased their daily total caloric intake on average by 1,200 kcal between the baseline and follow-up 1 visit (*P* < 0.05; Extended Data Table 1). No within-HCD group differences were observed between baseline and follow-up 2 visit (*P* < 0.05). Total caloric intake, including major macronutrient composition, did not differ between HCD and the control group at baseline and at the follow-up 2 study visit (no main effect of group or group-by-visit interaction; *P* > 0.05; Extended Data Table 1). There was a significant main effect of visit (baseline versus follow-up 2) for total caloric intake and all major macronutrient compositions (*P* < 0.05), with lower reported food intake at follow-up 2 compared with the baseline for both groups. State questionnaires on mood, desire to eat and food cravings showed no group differences across visits in the fasted state (Extended Data Table 2). Body weight and body composition did not differ between the groups and visits (*P* > 0.05; Table 1). However, liver fat content increased in the HCD group (group-by-visit interaction, estimate of −0.11, 95% CI −0.19 to −0.03, *P* = 0.008; HCD group baseline versus HCD follow-up 1, estimate of −0.3744, s.e. = 0.104, d.f. = 30.4, *t* = 3.6, *P* = 0.005; Extended Data Fig. 1), whereas it did not change in the control group (*P* = 0.958). No significant differences were identified for metabolic parameters (*P* > 0.05; Table 1), including peripheral insulin sensitivity based on the oral glucose tolerance test (oGTT)-derived Matsuda Index and the homeostasis model assessment insulin resistance (HOMA-IR), as well as inflammatory markers such as C-reactive protein (CRP), interleukin (IL)-6 (Table 1) and other cytokines (Extended Data Table 3).

**Table 1:** Participants’ metabolic characteristics

	Control		HCD	
	Baseline	Follow-up 1	Baseline	Follow-up 1
**Body composition**				
Body weight (kg)	73.9 ± 7.1	74.6 ± 7.0	72.3 ± 9.1	72.8 ± 9.4
BMI (kg m^−2^)	22.15 ± 1.4	22.35 ± 1.3	21.82 ± 2.4	21.95 ± 2.5
Waist-to-hip ratio	0.85 ± 0.1	0.86 ± 0.1	0.86 ± 0.1	0.86 ± 0.04
Total adipose tissue, MR-derived (l)	18.85 ± 5.7	18.16 ± 5.2	16.93 ± 4.7	17.43 ± 4.6
Subcutaneous adipose tissue, lower extremity, MR-derived (l)	8.74 ± 2.5	8.40 ± 2.3	8.04 ± 2.2	8.28 ± 2.1
Visceral adipose tissue, MR-derived (l)	1.60 ± 0.7	1.63 ± 0.6	1.70 ± 0.87	1.69 ± 0.9
Liver fat, ^1^H-MRS-derived (%)^a^	1.08 ± 0.6	1.22 ± 1.1	1.55 ± 2.2	2.54 ± 3.5
**Metabolic parameters**				
At follow-up 2: only fasting insulin, glucose, CRP and γ-glutamyl transferase were available				
	**Baseline**	**Follow-up 1**	**Baseline**	**Follow-up 1**
		**Follow-up 2**		**Follow-up 2**
HbA1c (%)	5.16 ± 0.2	5.15 ± 0.2	5.08 ± 0.3	5.09 ± 0.2
HbA1c (mmol mol^−1^)	32.82 ± 2.3	32.82 ± 2.5	32.12 ± 2.6	31.94 ± 1.9
Matsuda insulin sensitivity index (oGTT-derived)	18.15 ± 6.9	15.70 ± 5.7	18.12 ± 6.5	16.27 ± 6.2
HOMA-IR	1.9 ± 0.8	2.1 ± 0.7	1.7 ± 0.7	1.9 ± 0.7
		1.6 ± 0.9		1.7 ± 0.5
Fasting insulin (pmol l^−1^)	54.82 ± 22.5	61.09 ± 18.1	47.44 ± 17.8	54.67 ± 18.6
		46.60 ± 23.5		48.65 ± 14.3
Fasting glucose (mmol l^−1^)	4.68 ± 0.4	4.77 ± 0.4	4.77 ± 0.3	4.64 ± 0.3
		4.67 ± 0.3		4.61 ± 0.3
Fasting glucagon (pg ml^−1^)	84.18 ± 6.3	85.82 ± 7.3	85.78 ± 5.8	83.5 ± 5.7
Fasting triglycerides (mg dl^−1^)	69.18 ± 23.1	68.36 ± 29.1	65.61 ± 22.2	73.11 ± 33.1
IL-6-HS (pg ml^−1^)	1.08 ± 0.70	0.96 ± 0.62	1.01 ± 0.92	0.84 ± 0.43
CRP (mg l^−1^)	0.04 ± 0.06	0.07 ± 0.13	0.06 ± 0.08	0.05 ± 0.06
				0.10 ± 0.17
γ-Glutamyl transferase (U l^−1^)	21.00 ± 6.6	19.82 ± 7.3	21.39 ± 15.7	19.94 ± 13.1
				20.24 ± 15.9
Testosterone (nmol l^−1^)	17.49 ± 4.2	17.51 ± 4.5	19.18 ± 7.1	19.08 ± 5.2
				18.76 ± 5.4
**Indirect calorimetry**				
Resting energy expenditure (kcal)	2,115 ± 219	2,166 ± 216	2,195 ± 260	2,155 ± 275
Respiratory quotient	0.85 ± 0.1	0.89 ± 0.1	0.86 ± 0.1	0.88 ± 0.1

Data are presented as mean ± s.d.

^a^For liver fat, we observed a significant group-by-visit interaction of *P* = 0.008.

MR, magnetic resonance; HbA1c, glycated haemoglobin; MRS, magnetic resonance spectroscopy. *n* = 29.

**Fig. 2:**
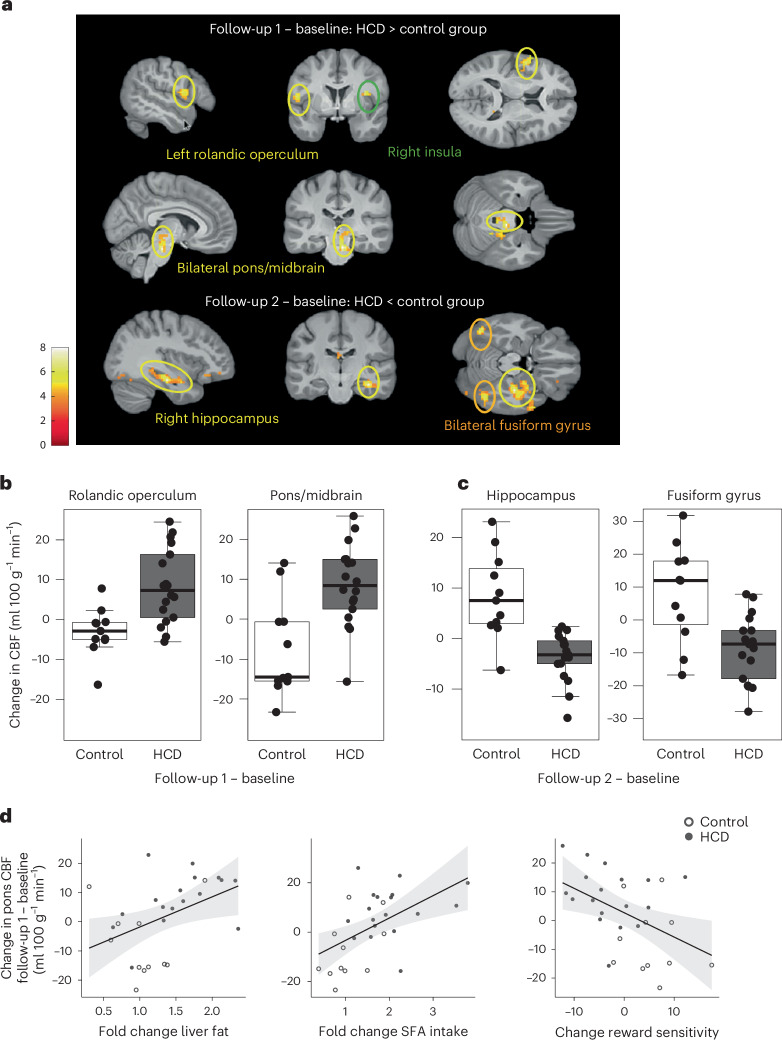
Disrupted brain insulin action after short-term overeating with calorie-rich snacks. **a**, Changes in brain insulin activity at follow-up 1 (directly after the 5-day HCD or regular diet) and follow-up 2 (1 week after resuming the regular diet) in HCD compared with the control group. Regions with significant changes in CBF after INI application in the HCD group compared with the control group and adjusted for the baseline measurement day are shown. Colour maps correspond to *t*-values (*P* < 0.001, uncorrected for display). **b**, Areas in the brain showing significantly higher insulin activity at follow-up 1 in the HCD compared with the control group adjusted for baseline measurement (*P*_FWE_ < 0.05, whole-brain cluster level corrected). *n* = 29 at baseline and follow-up 1. **c**, Areas in the brain showing significantly lower insulin activity at follow-up 2 in the HCD compared with the control group adjusted for baseline measurement day (*P*_FWE_ < 0.05, whole-brain cluster level corrected). *n* = 29 at baseline, *n* = 28 at follow-up 2. Box plots show at the centre the median values indicated by thick horizontal lines; upper and lower hinges correspond to first and third quartiles (25th and 75th percentiles). The whiskers extend from the hinges to the minimum and maximum value, which is 1.5 × interquartile range of the hinge. **d**, Higher brain insulin responsiveness at follow-up 1 (adjusted for the baseline visit) significantly correlated with the fold change in liver fat (*n* = 28; *r* = 0.434, *P* = 0.02), the food diary reported fold change in saturated fatty acid (SFA) intake (*n* = 29; *r* = 0.531, *P* = 0.003) and the change in reward sensitivity (*n* = 29) (*r* = −0.460, *P* = 0.01) at follow-up 1 adjusted for baseline. Source data

The primary aim of the study was to assess insulin-induced brain activity before (baseline), directly after the HCD (follow-up 1) and 1 week after a normal caloric diet was resumed (follow-up 2) compared with a control group maintaining their regular diet. The absolute change of cerebral blood flow (CBF) was used as a proxy for neural activity. We analysed the difference in brain insulin activity between the HCD and control groups at follow-up 1 and follow-up 2, adjusted for the individual baseline measurement (Fig. 2a). The HCD group had significantly higher insulin activity in parts of the right insular cortex, left rolandic operculum and right midbrain/pons (Fig. 2b) at follow-up 1 adjusted for baseline compared with the control group (*P*_FWE_ < 0.05, whole-brain corrected, where FWE indicates family wise error; Extended Data Table 4). At the second follow-up, 7 days after resuming the regular diet, the HCD group showed significantly lower brain insulin activity in the right hippocampus and bilateral fusiform gyrus compared with the control group (Fig. 2c; *P*_FWE_ < 0.05, whole-brain corrected; Extended Data Table 4). No differences were observed in hypothalamic response to insulin between the HCD and control groups at both follow-up time points (*P* > 0.05). At the baseline visit, no group differences were observed for the absolute change of CBF from before to after INI (*P*_FWE_ > 0.05). Independent of INI, we observed no differences between the HCD and the control group in regional CBF or global CBF (CBF before nasal spray application HCD versus controls; *P*_FWE_ > 0.05).

**Extended Data Fig. 2:**
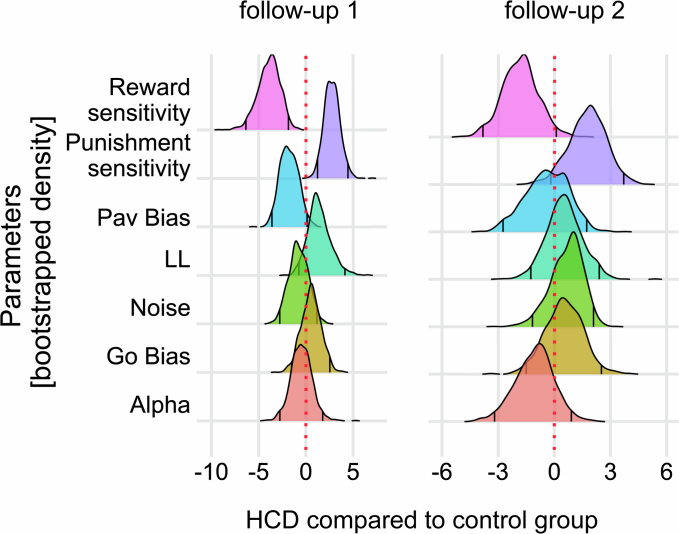
HCD reduced reward sensitivity and increased punishment sensitivity. Bootstrapped density plots of the difference between groups in changes of parameter estimates at follow-up 1 and follow-up 2 compared to baseline (pre), respectively. Lines indicate 95% confidence intervals. HCD = high-caloric diet group; RewS = Reward Sensitivity, Pav = Pavlovian, LL = log-likelihood. Source data

**Extended Data Fig. 3:**
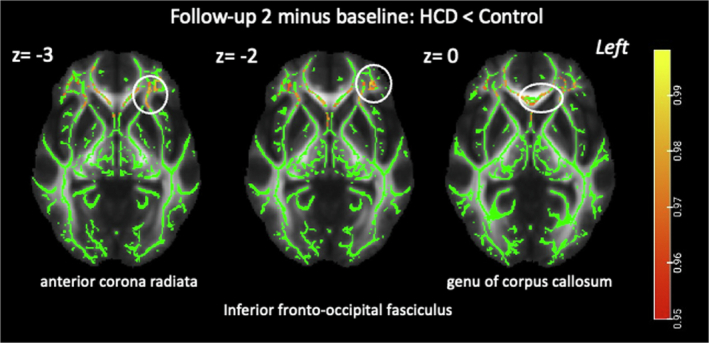
Change in white matter organization at follow-up 2. Change in white matter organization at follow-up 2 in the high-caloric diet compared to the control group. The panel shows the fractional anisotropy skeleton (in green) representing the major white matter tracts of all participants overlayed on a MNI standard brain. White matter fibre tracts showing a significantly lower fractional anisotropy (FA) at follow-up 2 in the high-caloric diet compared to the control group are shown in red, orange and yellow (p-corr <0.05). Lower FA values are mainly located on inferior fronto-occipital fasciculus, genu of the corpus callosum and anterior corona radiata (white circles). Colorbar represents 1-p value (tfce-corrected). Tracts in yellow displayed smaller p value or more significant results. N = 27. Source data

We further evaluated whether the HCD alters reward and punishment sensitivity. Compared with the control group, the HCD reduced reward sensitivity (*t*(27) = −3.6, *P*_boot_ < 0.001, where boot indicates bootstrapping) and increased punishment sensitivity (*t*(27) = 2.6, *P*_boot_ = 0.002) at follow-up 1 (Extended Data Fig. 2). Notably, this pattern was still evident at follow-up 2, although the effects on each parameter were not significant anymore (reward sensitivity, *t*(26) = −1.7, *P*_boot_ = 0.06; punishment sensitivity, *t*(26) = 1.7, *P*_boot_ = 0.07). Correlation analyses showed that higher insulin activity at follow-up 1 was significantly associated with fold change in liver fat, the change in reward learning and the food diary reported fold change in fat and saturated fatty acid intake, especially in the pons/midbrain (Fig. 2d) (*P* < 0.05; Extended Data Table 5). Lower insulin responsiveness in the fusiform gyrus 1 week after resuming a regular diet significantly correlated with the food diary reported fold change in carbohydrate intake and the hippocampal change in responsiveness to insulin correlated with the fold change in fat intake and saturated fatty acid intake (*P* < 0.05; Extended Data Table 5). Fractional anisotropy (FA) and mean diffusivity (MD) are summary measures of white matter diffusivity. The HCD group had significantly lower FA values mainly located on the inferior fronto-occipital fasciculus, genu of the corpus callosum and anterior corona radiata (Extended Data Fig. 3; *P* < 0.05; threshold-free cluster enhancement (TFCE)-corrected) as well as higher MD values in the superior corona radiata at follow-up 2 compared with baseline. No significant differences were observed at follow-up 1 compared with baseline. No group differences were observed at baseline for global FA values. However, significant group differences were observed for global MD values at baseline (*P* < 0.05). The control group showed higher MD values.

The current study demonstrates that brain insulin responsiveness adapts to short-term dietary changes after overconsumption of broadly available sweet and fatty ultra-processed snacks in addition to their regular diet, in healthy weight men, in the absence of changes in body weight, peripheral insulin sensitivity and food craving. Liver fat content, however, substantially increased after this HCD, which was directly related to the initially augmented response to brain insulin of food reward pathways. Moreover, reward learning was also disrupted as indicated by a decreased sensitivity for rewards and increased sensitivity for punishments. These initial alterations normalized 1 week after participants returned to their regular diet. Notably, insulin sensitivity of cognitive-related brain regions and markers of brain integrity were lower in the HCD compared with the control group after the HCD group resumed their regular diet. These data suggest that a short-term HCD, rich in sugar and saturated fat, has prolonged effects on the brain that outlast the time-frame of its consumption. Habitual daily intake of sweet and fatty snacks for 8 weeks has been shown to increase neural responses to food, while decreasing the preference for low-fat food independent of changes in body weight and metabolism^11^. In persons with obesity-associated insulin resistance, higher responsiveness to insulin has been observed in the insular cortex^12^ and midbrain^13^, similar to the increased response that we observed after HCD. Moreover, studies in people with obesity found poorer performance in dopamine-dependent reward-learning paradigms^14,15^. We could identify a similar alteration in the present study in healthy weight men after overconsumption of high-caloric food, providing evidence that altered reward learning and increased activity of the reward associated brain regions to insulin may be present before weight gain. After resuming a normal diet, we found a diminished response to brain insulin in the hippocampus and fusiform gyrus in the HCD group. Insulin activity in these regions plays an important role in attenuating the neural response to visual food cues^16^ and memory processes^3^. Under healthy conditions, INI improves performance on learning and memory tasks^3^ and increases hippocampal blood flow^17^ and hippocampal functional connectivity^18,19^. Of note, the diminished response to insulin in the hippocampus and fusiform gyrus outlasted the consumption of the HCD. Notably, hippocampal insulin resistance can develop independent of peripheral insulin sensitivity^20^, as identified in rodent models^20^ and in postmortem brain tissue of patients with Alzheimer’s disease^21^. Likewise, in the current study, diminished brain insulin responsiveness in the hippocampus was present without notable perturbations in peripheral metabolism, suggesting that diet-induced changes in brain insulin responsiveness precede peripheral insulin resistance. Together, these findings add a novel facet to our understanding of the possible development of brain insulin resistance and suggest that the detrimental effects of a HCD persists even after cessation of the unhealthy nutritional stimulus. Initial evidence indicates that, particularly in women, hippocampal insulin sensitivity decreases with age^17^. Whether our findings can be extended to women needs to be investigated in future studies. Besides insulin resistance, there are a multitude of factors that substantially contribute to the pathophysiology of obesity. Particularly, abdominal obesity is a source of inflammation^22^ and dietary excess can trigger inflammation in the brain in the preclinical model, involving non-neural populations such as astrocytes, microglia and tanycytes in the hypothalamus^23,24^ as well as the orbitofrontal cortex^25^. While less is known about inflammation in the brain in humans, initial imaging studies point to structures of the hypothalamus and reward pathways being vulnerable to obesity-associated inflammation^26,27^. Likewise, in the current study, we found reductions in white matter integrity between reward and cognitive regions after participants resumed their regular diet, similar to changes in individuals with obesity^28^. Based on the nature of the MRI signal, these alterations in white matter integrity are based on changes in brain water content, which is known to be mediated by obesity-associated inflammation^26^. Whether systemic inflammation contributed to these changes cannot be answered in the current study. However, no changes in cytokines were observed immediately after the HCD. Clearly, further studies are needed to disentangle the role of brain insulin responsiveness and inflammation in the development of obesity and associated diseases, as subclinical low-grade inflammation is well known to affect metabolic regulation.

In conclusion, we show that short-term overeating with commonly used ultra-processed high-caloric snacks can trigger liver fat accumulation and short-term disrupted brain insulin action that outlast the time-frame of the HCD in men. We postulate that the brain response to insulin adapts to short-term changes in diet before weight gain and may facilitate the development of obesity and associated diseases.

The small sample size of our study limits the generatability of our results and may be the reason why we did not identify impairments in peripheral insulin sensitivity after the 5-day HCD. Furthermore, we did not investigate whole-body insulin sensitivity based on the gold standard (hyperinsulinaemic euglycaemic clamp). Knudsen et al.^29^ were able to observe changes in whole-body insulin sensitivity after just 3 days of overeating and physical inactivity, before changes in body weight^29^. This could indicate that inactivity facilitates a more rapid decrease in peripheral insulin sensitivity than the HCD alone. Furthermore, with the current study design, we cannot disentangle the effect of excessive calories on the brain versus the influence of excessive calories of specific macronutrients. Whether excessive intake of healthy calories or physical inactivity impacts brain insulin activity is the subject of future studies. For follow-up 2, no liver fat measurement and no blood sampling during oGTT were available. Hence, we cannot conclude whether the liver fat accumulation, inflammation or free fatty acid species play an important role beyond the HCD. Also, the study duration was too short to evaluate long-term effects of the HCD. We investigated the effect of HCD exclusively in male participants. Sex-specific findings of insulin action on appetite, metabolism and memory have been reported^3^, which depend in part on the menstrual cycle^10^. It is likely that adaptations of brain insulin activity to diet additionally depend on hormonal fluctuations in women.

## Methods

### Participants

Twenty-nine male participants were aged 19–27 years, were healthy weight (BMI 19–25 kg m^−2^), nonsmokers, of stable weight for at least 3 months before the study visits, nondieters, not vegan or vegetarian dieters, without food allergies, exercising less than 2 h a week, not working at night, not taking medication, and with no history of diabetes, eating disorders, illicit drug use or other medical diagnoses. Gender was determined based on self-report and supported by testosterone measurements. In a nonrandomized controlled design, 18 participants were enrolled to participate in a 5-day HCD (snacks in addition to their regular diet) and 11 participants were enrolled as the control group (to maintain their regular diet with no additional calories). Data collection and analysis were not performed blind to the conditions of the experiments. Participants provided written informed consent in compliance with the University of Tübingen ethical committee. The study (813/2017BO2) received approval by the local ethics committee in January 2018 (Ethics Committee of the Medical Faculty of the Eberhard Karls University and the University Hospital Tübingen) and was conducted according to the relevant guidelines and regulations. The study is a basic experimental study involving humans and was registered at ClinicalTrials.gov (NCT03590561). A compensation of €600 was provided after completion of the study (total of 23 h over six visits).

A previous study investigating brain insulin action^6^, showed a large effect size of Cohen’s *d* > 1 could be achieved with 18 participants per group to detect obesity-associated insulin resistance (healthy weight versus obese). Assuming a two-sided *t*-test of *α* = 0.05 and 80% power, a sample size of at least 25 people was calculated for the total sample size.

### Study overview

Brain insulin action was assessed by fMRI combined with intranasal administration of insulin to the brain before, directly after the HCD or regular diet, and 1 week afterwards. The timing of the follow-up visits was adapted to the first day of the consumption of the HCD. Food intake and physical activity were recorded during the course of the study. Moreover, participants underwent two five-point 75 g oGTTs according to the methods of Matsuda and DeFronzo^30^ to assess peripheral insulin sensitivity, whole-body MRI for body fat distribution/intrahepatic fat content, and performed a reward-learning task (see Fig. 1 for study overview). For each study visit, participants arrived at the study centre at ~08:00 after an overnight fast of 12 h. Blood samples were taken during the oGTT and once before each fMRI measurement. Participants were instructed by a nutritionist to record their food intake into a diary and provide pictures of all their meals and instructed to walk fewer than 4,000 steps a day, monitored using a Fitbit watch (Fitbit Inspire or Fitbit Inspire HR; Fitbit LLC, USA). High-caloric snacks were provided to the participants based on palatability ratings obtained before the baseline visit. Only participants who habitually consumed high-caloric snacks (at least four times a week) were included.

### High-caloric diet

Based on the participant specific palatability ratings, a nutritionist prepared packages for 5 days containing 1,500 kcal each with different snacks (including for example, Snickers, brownies and chips, with a nutritional composition equivalence of 47–50% fat and 40–45% carbohydrates; see Supplementary Table 1).

### Food diaries

Participants in the HCD group completed food diaries on three consecutive days before the intervention, five consecutive days during the intervention, and again three consecutive days at the end of the assessment period before follow-up 2. The control group completed food diaries for three consecutive days at two time points corresponding to the time between the baseline/follow-up 1 and follow-up 2 visits. Diet composition was estimated with a validated software (DGE-PC 3.0; German Nutrition Society). In addition, all consumed food was photographed by the participants to validate the information provided in the diary.

### Whole-body MRI for quantification of adipose tissue compartments

T1-weighted fast spin-echo images with a 1-cm slice thickness and an interslice gap of 1 cm were acquired from the entire body on a 3T whole-body scanner (Magnetom Vida; Siemens Healthineers) in the early morning after overnight fasting^31^. After MRI, single-voxel MR spectroscopy was performed in the posterior part of segment 7 of the liver for quantification of IHL, which was calculated by the ratio of lipid signal and water plus lipid signal^32^.

### Whole-brain MRI measurement and preprocessing

Scanning was conducted at a 3T whole-body Siemens scanner (Magnetom Prisma) with a 20-channel head/neck coil. Brain insulin responsiveness was quantified by application of INI in combination with fMRI recordings. Measurements were performed under fasting conditions. After the basal measurement, 160 U insulin spray was administered intranasally (Insulin Actrapid; Novo Nordisk). After 30 min, a second fMRI measurement was performed. To acquire CBF maps, pulsed arterial spin labelling images with a PICORE-Q2TIPS sequence (proximal inversion with control for off-resonance effects—quantitative imaging of perfusion by using a single subtraction) using a frequency offset corrected inversion pulse and echo planar imaging readout for acquisition^6,33^. In addition, high-resolution T1-weighted anatomical images were obtained. Image preprocessing was performed using the ASLtbx with SPM12 (Wellcome Trust Centre for Neuroimaging). Functional images were motion corrected, co-registered to the individual anatomical image and smoothed (full width half maximum 6 mm). Perfusion images were generated by calculating the control-tag differences by using surround subtraction. For accurate CBF quantification (ml 100 g^−1^ min^−1^), we used unique M0 values extracted from a region of interest in the cerebrospinal fluid^34^. We used the general kinetic model for absolute perfusion quantification. Recent reliability studies and our measurements show high reproducibility and reliability for CBF maps^34,35^.

Diffusion-weighted images were acquired at each visit before INI administration, to investigate white matter integrity. An echo planar imaging sequence (70 axial slices, field of view of 220 mm^2^, slice thickness of 2 mm, TE = 54 ms and TR = 6,500 ms) with 35 directions (*b* = 1,000 s mm^−2^) and GRAPPA acceleration factor 2 was acquired. Moreover, 11 interspersed nondiffusion weighted volumes (b0) were recorded. To improve signal-to-noise ratio two averages were performed. The measurement lasted a total of 8 min 3 s. Standard preprocessing and statistical analyses were performed using FMRIB (Functional Magnetic Resonance Imaging of the Brains, Oxford University) Software Library (FSL v.6.0). For all FA images, a nonlinear registration in MNI152 space was conducted using the FMRIB58_FA template as target image. The mean FA image pooled over all participants and sessions was computed and the skeleton representing the main white matter tracts was created. Thereafter, the aligned four-dimensional skeletonized FA images were thresholded (FA ≥ 0.2) to reduce partial volume effects. For the nonlinear registration of the MD images into MNI space, the transformation parameters of the FA images were applied and skeletonized. One participant was excluded from the analyses because of imperfect slice positioning cutting parts of the white matter tracts.

### Statistics

Unless otherwise stated, data are presented as mean ± s.d. The primary analysis was to assess insulin-induced changes in brain activity before (baseline) and twice after the intervention (follow-up 1 and follow-up 2). To this end, the absolute change of CBF of each participant before and after INI spray application was used for further statistics (∆CBF, CBF 30 min after nasal spray − CBF before nasal spray). Whole-brain analyses were performed using a voxel-wise approach in SPM12. ∆CBF of the baseline measurement day was subtracted from the follow-up visits (∆CBF_follow-up 1/2_ − ∆CBF_baseline_) and entered into a flexible factorial design to investigate the difference in brain insulin responsiveness between the HCD and control groups at follow-up 1 and follow-up 2, while taking into account within-subject variability (within-subject factors, subject ID and visit (follow-up 2 and follow-up 1); between-subject factor, group (HCD and control)). A statistical threshold of *P* < 0.001 uncorrected and *P*_FWE _< 0.05 cluster level corrected for multiple comparisons was applied on a whole-brain level.

To investigate differences in white matter integrity between the HCD and control group, FA and MD values from the baseline to follow-up 1 and follow-up 2 (follow-up 1/2 − baseline) were calculated for each participant on the skeletonized data using fslmaths. A two-sample unpaired *t*-test was performed between the HCD and the control group for FA and MD values, respectively. We included the global FA/MD values as a covariate of no interest. For tract-based spatial statistics, we used the module randomize in FSL, which is a nonparametric permutation test for inference on statistical maps^36^. For detecting significant clusters corrected for multiple comparisons, we selected the TFCE method optimized for TBSS analyses^37^. Statistical maps were thresholded at *P* < 0.05 (TFCE corrected). The number or permutations was set to 5,000. Tract labels were assigned using JHU ICBM-DTI-81 white matter labels or the HCP white matter probability tracts.

Secondary analyses were conducted using R (v.4.3.3.). Groups, visits as well as group-by-visit interactions were tested by linear mixed-effects models employing the lmer function of the lme4 package in R. Participants were included as random factors to account for within-subject correlations. Groups and visits were considered as fixed effects. Normal distribution was investigated by visual inspection and QQ plots. In exploratory analyses, correlations were used to associate metabolic and behavioural changes with changes in brain insulin action (*P* < 0.05 uncorrected for multiple comparisons).

### Questionnaires

Participants reported impulsivity (Barratt impulsiveness scale^38^), eating behaviour (food craving questionnaire – trait)^39^ and the three-factor eating questionnaire^40^; German version, fragebogen zum essverhalten^41^). Furthermore, eating disorders (eating disorder examination^42^), depression (Becks-depressions-inventar^43^) and psychiatric disease (patient health questionnaire^44^) were ruled out. Before and after the INI, participants rated their mood (positive and negative affect schedule questionnaire^45^) and their subjective feeling of hunger on a visual analogue scale (0, not hungry at all to 10, very hungry).

### Go/no-go learning task

We investigated reward learning with an established valence-dependent go/no-go learning paradigm^46^. Participants were asked to learn correct approach (go) or inhibitory (no-go) responses to cues that predicted reward or punishment. With this task, participants learned state–action contingencies and received rewards or punishments. Each trial consisted of three stages; 240 trials in total, consisting of 120 go trials and 120 no-go trials, each with 60 trials for each condition (for example, win or avoid loss); the task duration was 15 min. Using a laptop computer, fractal cues (state) were presented out of a set of four different fractals per session. Fractals were randomized to one of the four possible combinations of the go × win two-factorial design of the task. Participants completed a target detection task and either respond by pressing a key (go) or withhold their response (no-go). The outcome of the state–action combination is visualized on the screen, which was either a win (5 cents), punishment (−5 cents) or an omission (no win/punishment, 0 cents). Using trial and error, participants learned which action following each fractal was best in terms of maximizing wins or minimizing losses. Outcomes were presented probabilistically, as follows: 80% chance to win after correct state–action sequences; 20% chance to win after incorrect sequences for rewarded trials; 80% chance to avoid losses after correct; and 20% chance to avoid losses after incorrect sequences for punished trials. Participants were told about the probabilistic nature of the task and that either go or no-go responses could be correct for a given fractal. There was no change in the contingencies over time. To ensure that participants understood the task, they performed a practice run at the beginning.

### Reinforcement learning model

We used computational modelling to test the effects of the HCD on specific processes of value-based decision-making. To this end, we fit previously reported reinforcement learning models that are extensions of standard Q-learning models^46^. In brief, specific action (*a*) values (*Q*) are updated for the shown stimulus (*s*) at each trial *t* with the Rescorla–Wagner rule:

$${Q}_{t}({s}_{t},{a}_{t})={Q}_{t-1}({s}_{t},{a}_{t})+\alpha (\rho {r}_{t}-{Q}_{t-1}({s}_{t},{a}_{t})).$$

The speed of learning is modified by the learning rate *α* (*α* ϵ [0,1]) and individual reward sensitivity is scaled by *ρ*, a free, positive parameter. Obtained rewards *r*_*t*_ are coded as −1, 1 or 0 if participants received a punishment, reward or neither, respectively. To account for Pavlovian effects on learning, action-independent values (*V*) for each stimulus are learned with the same Rescorla–Wagner rule to indicate whether a stimulus leads to punishment or reward:

$$V({s}_{t})={V}_{t-1}({s}_{t})+\alpha (\rho {r}_{t}-{V}_{t-1}({s}_{t}))$$

Both action values (*Q*) and stimulus values (*V*) are then combined to action weights (*W*) at each trial:

$${W}_{t}(a,s)=\left\{\begin{array}{l}{Q}_{t}\left(a,s\right)+b+\pi {V}_{t}(s),\qquad{a=\rm{go}}\\\qquad\qquad\qquad {Q}_{t}(a,s),\qquad\rm{else}\end{array}\right.$$

Here, *b* (>0) reflects the constant tendency to choose the go option and Pavlovian tendencies are parameterized by *π*, a positive free parameter that captures impaired learning in trials with incongruent Pavlovian and instrumental behaviour (for example, go-to-avoid punishment). Last, the action probability (*p*) at each trial is determined using a softmax function where a noise parameter ($\,\xi \,\in \left[\mathrm{0,1}\right]$) scales how much influence learned values have on decisions:

$$p\left({a}_{t}|{s}_{t}\right)=\left[\frac{\exp \left(W\left({a}_{t}|{s}_{t}\right)\right)}{{\sum }_{a}\exp (W\left({a}^{{\prime} }|{s}_{t}\right))}\right]\times \xi +\left(\frac{1-\xi }{2}\right)$$

In addition, we fit two further models disentangling valence specific effects by estimating either reward sensitivity, or reward sensitivity and learning rate separately for reward and punishment. To test whether valence effects were specific for reward sensitivity versus learning rates^47^, we also explored a model that separated learning rates, but not reward sensitivity based on outcome valence. As the model that included a valenced learning rate did not converge, it precluded further analyses. As previously described, we used hierarchical expectation maximization to fit the models^48^. Using this method, individual parameters and the group distributions of the parameters are estimated iteratively, so that at each iteration, the current group-level distributions are used as priors for individual parameter estimation (Laplace approximation). Consequently, group-level distributions are updated integrating the new individual estimates and their uncertainty. To avoid systematic bias, we treated each repeated sessions (baseline, follow-up 1 and follow-up 2) as an independent measurement. To prevent exaggerating differences between both groups, we fit one underlying distribution over all participants and repeated measurements. We compared models using group-level integrated BIC (iBIC^48^), which combines model fit and model complexity across all measurements. To constrain parameters to their theoretical range, we log-transformed reward sensitivity and Pavlovian bias and used an inverse sigmoid transformation for the learning rate and irreducible noise.

We evaluated intervention effects by comparing changes in parameter estimates between the HCD and the control group using independent-sample *t*-tests with a significance threshold of *P* < 0.05 (two-tailed). As model parameters might not be normally distributed, we used bootstrapping with 1,000 samples to compare changes in parameter estimates at follow-up 1 and follow-up 2 between groups. Moreover, we corrected for multiple comparisons across the six parameters in the winning computational model analysis using Bonferroni correction. We performed data analyses using MATLAB v.2016a (computational model).

To evaluate whether HCD alters reward and punishment sensitivity, we fitted computational reward-learning models^46^ that dissociate sensitivity in response to wins or losses as outcomes. In line with Guitart-Masip et al.^49^, a model including six parameters with valenced (reward/punishment) reward sensitivities provided a better model fit compared with the standard five-parameter model (∆iBIC = −184). In this model, HCD had opposing effects on reward and punishment sensitivity at both post intervention time points (Extended Data Fig. 2). We also explored a model only including a valenced learning rate but not a valenced reward sensitivity as previously described^47^. However, while this model provided a slightly better model fit compared with the winning model (∆iBIC = −74) at the group level, it only provided a more parsimonious account at the individual (iBIC) level for 23 out of 86 runs. Nonetheless, there were no significant differences between groups at both times, neither for the single reward sensitivity nor the valenced learning rates, indicating that effects were specific for reward sensitivity.

### Detection of cytokines

Cytokines were quantified using a combination of Bio-Plex Pro Human Cytokine Plex Panels (Luminex; Bio-Rad Laboratories). Multiplex assay was performed on a Luminex 200 system in accordance with the manufacturer’s instructions. Samples were measured at two different time points on different plates. Hence, values were time-point-normalized for statistical analyses.

### Reporting summary

Further information on research design is available in the Nature Portfolio Reporting Summary linked to this article.

## Supplementary information

Supplementary Information Supplementary Table 1 and Consort file diagram.

Reporting Summary

## Source data

Source Data Fig. 2 Statistical source data for fMRI data.

Source Data Table 1 Participants’ metabolic characteristics.

Source Data Extended Data Fig. 1 Statistical source data of liver fat content.

Source Data Extended Data Fig. 2 Statistical source data of reward and punishment sensitivity.

Source Data Extended Data Fig. 3 Link to Neurovault platform for diffusion imaging statistical data.

Source Data Extended Data Table 1 Statistical source data of food diary.

Source Data Extended Data Table 2 Statistical source data of state questionnaires.

Source Data Extended Data Table 3 Statistical source data of time-point-normalized inflammatory markers.

Source Data Extended Data Table 4 Link to Neurovault for brain imaging collection.

Source Data Extended Data Table 5 Statistical source data of correlation analyses.

## Figures and Tables

**Extended Data Table 1:**
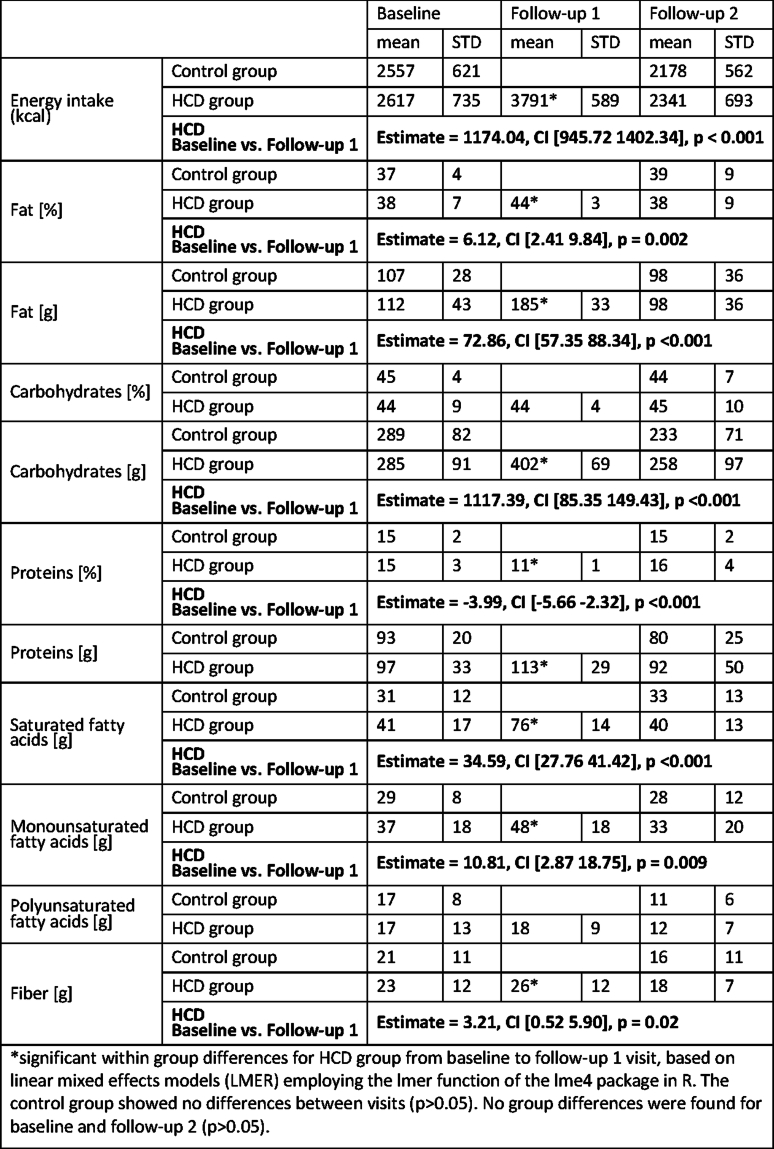
Food Diary

**Extended Data Table 2:**
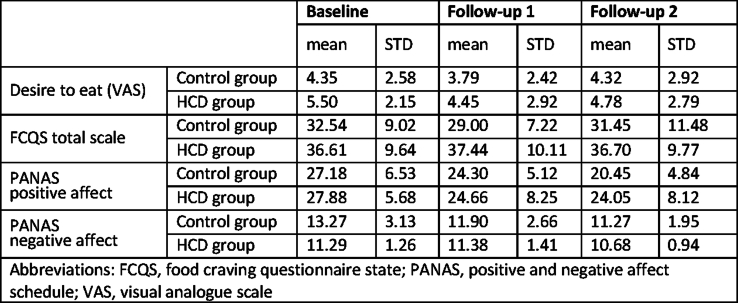
State Questionnaires on Brain MRI day in the fasted state

**Extended Data Table 3:**
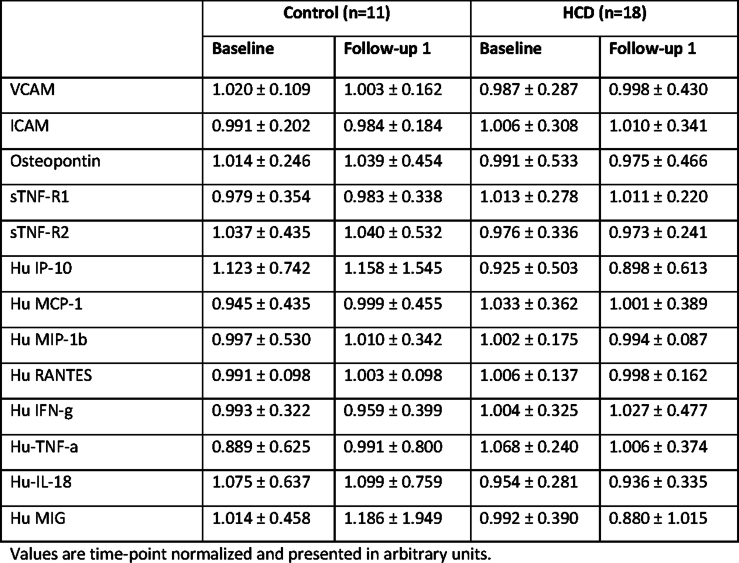
Inflammatory markers

**Extended Data Table 4:**
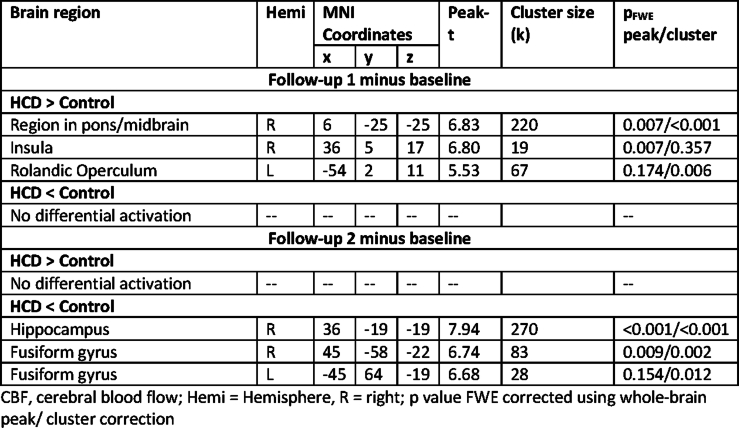
Changes in brain insulin activity (CBF) from before to after the intervention

**Extended Data Table 5:**
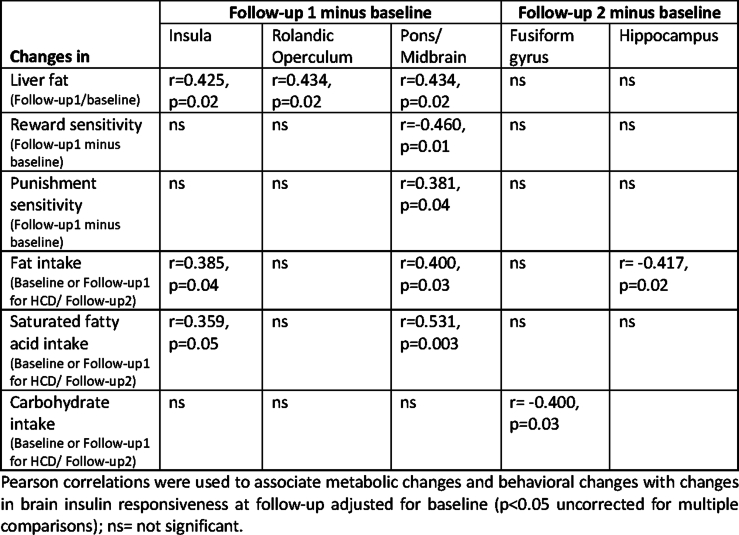
Correlation between change in brain insulin responsiveness and metabolic and behavioural changes

## Data Availability

Statistical maps of the brain effects are uploaded to Neurovault (https://neurovault.org/collections/MYQHVMQE/). Source data are provided with this paper.

## Code availability

No custom code was used.

## References

[1] DeFronzo, R. A. et al. Type 2 diabetes mellitus. *Nat. Rev. Dis. Primers* **1**, 15019 (2015). 27189025 10.1038/nrdp.2015.19

[2] Kullmann, S. et al. Central nervous pathways of insulin action in the control of metabolism and food intake. *Lancet Diabetes Endocrinol.* **8**, 524–534 (2020). 32445739 10.1016/S2213-8587(20)30113-3

[3] Hallschmid, M. Intranasal insulin. *J. Neuroendocrinol.* **33**, e12934 (2021). 33506526 10.1111/jne.12934

[4] Kellar, D. & Craft, S. Brain insulin resistance in Alzheimer’s disease and related disorders: mechanisms and therapeutic approaches. *Lancet Neurol*. **19**, 758–766 (2020). 32730766 10.1016/S1474-4422(20)30231-3 PMC9661919

[5] Scherer, T., Sakamoto, K. & Buettner, C. Brain insulin signalling in metabolic homeostasis and disease. *Nat. Rev. Endocrinol.* **17**, 468–483 (2021). 34108679 10.1038/s41574-021-00498-x

[6] Kullmann, S. et al. Selective insulin resistance in homeostatic and cognitive control brain areas in overweight and obese adults. *Diabetes Care* **38**, 1044–1050 (2015). 25795413 10.2337/dc14-2319

[7] Kullmann, S. et al. Brain insulin sensitivity is linked to adiposity and body fat distribution. *Nat. Commun.* **11**, 1841 (2020). 32296068 10.1038/s41467-020-15686-y PMC7160151

[8] Rebelos, E., Nummenmaa, L., Dadson, P., Latva-Rasku, A. & Nuutila, P. Brain insulin sensitivity is linked to body fat distribution-the positron emission tomography perspective. *Eur. J. Nucl. Med. Mol. Imaging* **48**, 966–968 (2021). 33029655 10.1007/s00259-020-05064-7 PMC8041695

[9] Gruber, J. et al. Impact of insulin and insulin resistance on brain dopamine signalling and reward processing - An underexplored mechanism in the pathophysiology of depression? *Neurosci. Biobehav. Rev.* **149**, 105179 (2023). 37059404 10.1016/j.neubiorev.2023.105179

[10] Hummel, J. et al. Brain insulin action on peripheral insulin sensitivity in women depends on menstrual cycle phase. *Nat. Metab*. **5**, 1475–1482 (2023). 37735274 10.1038/s42255-023-00869-w PMC10513929

[11] Edwin Thanarajah, S. et al. Habitual daily intake of a sweet and fatty snack modulates reward processing in humans. *Cell. Metab.* **35**, 571–584 e576 (2023). 36958330 10.1016/j.cmet.2023.02.015

[12] Wingrove, J. O. et al. Intranasal insulin administration decreases cerebral blood flow in cortico-limbic regions: a neuropharmacological imaging study in normal and overweight males. *Diabetes Obes. Metab.* **23**, 175–185 (2021). 33026175 10.1111/dom.14213

[13] Tiedemann, L. J. et al. Central insulin modulates food valuation via mesolimbic pathways. *Nat. Commun.* **8**, 16052 (2017). 28719580 10.1038/ncomms16052 PMC5520049

[14] Kroemer, N. B. & Small, D. M. Fuel not fun: reinterpreting attenuated brain responses to reward in obesity. *Physiol. Behav.* **162**, 37–45 (2016). 27085908 10.1016/j.physbeh.2016.04.020 PMC4971522

[15] Gill, H. et al. The impact of overweight/obesity on monetary reward processing: a systematic review. *J. Psychiatr. Res.* **137**, 456–464 (2021). 33798972 10.1016/j.jpsychires.2021.03.029

[16] Guthoff, M. et al. Insulin modulates food-related activity in the central nervous system. *J. Clin. Endocrinol. Metab.* **95**, 748–755 (2010). 19996309 10.1210/jc.2009-1677

[17] Wagner, L. et al. Brain insulin responsiveness is linked to age and peripheral insulin sensitivity. *Diabetes Obes. Metab.* **25**, 2171–2180 (2023). 37046367 10.1111/dom.15094

[18] Kullmann, S. et al. Intranasal insulin enhances brain functional connectivity mediating the relationship between adiposity and subjective feeling of hunger. *Sci. Rep.* **7**, 1627 (2017). 28487570 10.1038/s41598-017-01907-w PMC5431641

[19] Zhang, H. et al. Intranasal insulin enhanced resting-state functional connectivity of hippocampal regions in type 2 diabetes. *Diabetes* **64**, 1025–1034 (2015). 25249577 10.2337/db14-1000 PMC4338591

[20] Grillo, C. A., Woodruff, J. L., Macht, V. A. & Reagan, L. P. Insulin resistance and hippocampal dysfunction: disentangling peripheral and brain causes from consequences. *Exp. Neurol.* **318**, 71–77 (2019). 31028829 10.1016/j.expneurol.2019.04.012

[21] Talbot, K. et al. Demonstrated brain insulin resistance in Alzheimer’s disease patients is associated with IGF-1 resistance, IRS-1 dysregulation, and cognitive decline. *J. Clin. Invest.* **122**, 1316–1338 (2012). 22476197 10.1172/JCI59903 PMC3314463

[22] Alexopoulos, N., Katritsis, D. & Raggi, P. Visceral adipose tissue as a source of inflammation and promoter of atherosclerosis. *Atherosclerosis* **233**, 104–112 (2014). 24529130 10.1016/j.atherosclerosis.2013.12.023

[23] Garcia-Caceres, C. et al. Role of astrocytes, microglia, and tanycytes in brain control of systemic metabolism. *Nat. Neurosci.* **22**, 7–14 (2019). 30531847 10.1038/s41593-018-0286-y

[24] Porniece Kumar, M. et al. Insulin signalling in tanycytes gates hypothalamic insulin uptake and regulation of AgRP neuron activity. *Nat. Metab.* **3**, 1662–1679 (2021). 34931084 10.1038/s42255-021-00499-0 PMC8688146

[25] Lau, B. K. et al. Obesity-induced astrocyte dysfunction impairs heterosynaptic plasticity in the orbitofrontal cortex. *Cell Rep.* **36**, 109563 (2021). 34407401 10.1016/j.celrep.2021.109563

[26] Kullmann, S. et al. Investigating obesity-associated brain inflammation using quantitative water content mapping. *J. Neuroendocrinol.* **32**, e12907 (2020). 33025697 10.1111/jne.12907

[27] Dorfman, M. D. & Thaler, J. P. Hypothalamic inflammation and gliosis in obesity. *Curr. Opin. Endocrinol. Diabetes Obes.* **22**, 325–330 (2015). 26192704 10.1097/MED.0000000000000182 PMC4600090

[28] Daoust, J. et al. White matter integrity differences in obesity: a meta-analysis of diffusion tensor imaging studies. *Neurosci. Biobehav. Rev.* **129**, 133–141 (2021). 34284063 10.1016/j.neubiorev.2021.07.020

[29] Knudsen, S. H. et al. Changes in insulin sensitivity precede changes in body composition during 14 days of step reduction combined with overfeeding in healthy young men. *J. Appl. Physiol.* **113**, 7–15 (2012). 22556394 10.1152/japplphysiol.00189.2011

[30] Matsuda, M. & DeFronzo, R. A. Insulin sensitivity indices obtained from oral glucose tolerance testing: comparison with the euglycemic insulin clamp. *Diabetes Care* **22**, 1462–1470 (1999). 10480510 10.2337/diacare.22.9.1462

[31] Wurslin, C. et al. Topography mapping of whole body adipose tissue using a fully automated and standardized procedure. *J. Magn. Reson. Imaging* **31**, 430–439 (2010). 20099357 10.1002/jmri.22036

[32] Machann, J. et al. Hepatic lipid accumulation in healthy subjects: a comparative study using spectral fat-selective MRI and volume-localized 1H-MR spectroscopy. *Magn. Reson. Med.* **55**, 913–917 (2006). 16506186 10.1002/mrm.20825

[33] Kullmann, S. et al. Empagliflozin improves insulin sensitivity of the hypothalamus in humans with prediabetes: a randomized, double-blind, placebo-controlled, phase 2 trial. *Diabetes Care* **45**, 398–406 (2022). 34716213 10.2337/dc21-1136 PMC8914418

[34] Kullmann, S. et al. Exercise restores brain insulin sensitivity in sedentary adults who are overweight and obese. *JCI Insight* **7**, e161498 (2022). 36134657 10.1172/jci.insight.161498 PMC9675563

[35] Ssali, T. et al. Mapping long-term functional changes in cerebral blood flow by arterial spin labeling. *PLoS ONE* **11**, e0164112 (2016). 27706218 10.1371/journal.pone.0164112 PMC5051683

[36] Winkler, A. M., Ridgway, G. R., Webster, M. A., Smith, S. M. & Nichols, T. E. Permutation inference for the general linear model. *Neuroimage* **92**, 381–397 (2014). 24530839 10.1016/j.neuroimage.2014.01.060 PMC4010955

[37] Smith, S. M. & Nichols, T. E. Threshold-free cluster enhancement: addressing problems of smoothing, threshold dependence and localisation in cluster inference. *Neuroimage* **44**, 83–98 (2009). 18501637 10.1016/j.neuroimage.2008.03.061

[38] Meule, A., Vögele, C. & Kübler, A. Psychometrische evaluation der Deutschen Barratt Impulsiveness Scale-Kurzversion (BIS-15). *Diagnostica* **57**, 126–133 (2011).

[39] Meule, A., Hermann, T. & Kubler, A. A short version of the Food Cravings Questionnaire-Trait: the FCQ-T-reduced. *Front. Psychol.* **5**, 190 (2014). 24624116 10.3389/fpsyg.2014.00190 PMC3940888

[40] Stunkard, A. J. & Messick, S. The three-factor eating questionnaire to measure dietary restraint, disinhibition and hunger. *J. Psychosom. Res.* **29**, 71–83 (1985). 3981480 10.1016/0022-3999(85)90010-8

[41] Pudel, V. & Westenhöfer, J. *Fragebogen zum Essverhalten (FEV): Handanweisung* (Verlag für Psychologie Hogrefe, 1989).

[42] Hilbert, A. & Tuschen-Caffier, B. *Eating Disorder Examination: Deutschsprachige Übersetzung* (Verlag für Psychotherapie, 2006).

[43] Hautzinger, M., Keller, F. & Kühner, C. *BDI-II. Beck Depressios Inventar Revision-Manual* (Hartcourt Test Services, 2006).

[44] Löwe, B., Spitzer, R.L., Zipfel, S. & Herzog, W. *PHQ-D* (Gesundheitsfragebogen für Patienten, 2002).

[45] Krohne, H. W., Egloff, B., Kohlmann, C.-W. & Tausch, A. Untersuchung der deutschen Form der Positiven und Negativen Affect Schedule (PANAS). *Diagnostica* **42**, 139–156 (1996).

[46] Guitart-Masip, M. et al. Go and no-go learning in reward and punishment: interactions between affect and effect. *Neuroimage* **62**, 154–166 (2012). 22548809 10.1016/j.neuroimage.2012.04.024 PMC3387384

[47] Kuhnel, A. et al. Stimulation of the vagus nerve reduces learning in a go/no-go reinforcement learning task. *Eur. Neuropsychopharmacol.* **35**, 17–29 (2020). 32404279 10.1016/j.euroneuro.2020.03.023

[48] Huys, Q. J. et al. Disentangling the roles of approach, activation and valence in instrumental and pavlovian responding. *PLoS Comput. Biol.* **7**, e1002028 (2011). 21556131 10.1371/journal.pcbi.1002028 PMC3080848

[49] Guitart-Masip, M. et al. Differential, but not opponent, effects of L-DOPA and citalopram on action learning with reward and punishment. *Psychopharmacology* **231**, 955–966 (2014). 24232442 10.1007/s00213-013-3313-4 PMC3923110

[50] Preissl, H. Figure 1. *BioRender* https://BioRender.com/d74p651 (2025).

