# Supplementary material for: A short-term, high-caloric diet has prolonged effects on brain insulin action in men
Source: Nat Metab. 2025 Feb 21;7(3):469–77. doi: 10.1038/s42255-025-01226-9 (PMC11946887; doi:10.1038/s42255-025-01226-9)
Supplement: Supplementary file 1 — Supplementary Table 1 and Consort file diagram. [file 42255_2025_1226_MOESM1_ESM.pdf]

nature metabolism

Supplementary information https://doi.org/10.1038/s42255-025-01226-9

A short-term, high-caloric diet has prolonged effects on brain insulin action in men

In the format provided by the authors and unedited

CONSORT
TRANSPARENT REPORTING of TRIALS

CONSORT 2010 Flow Diagram

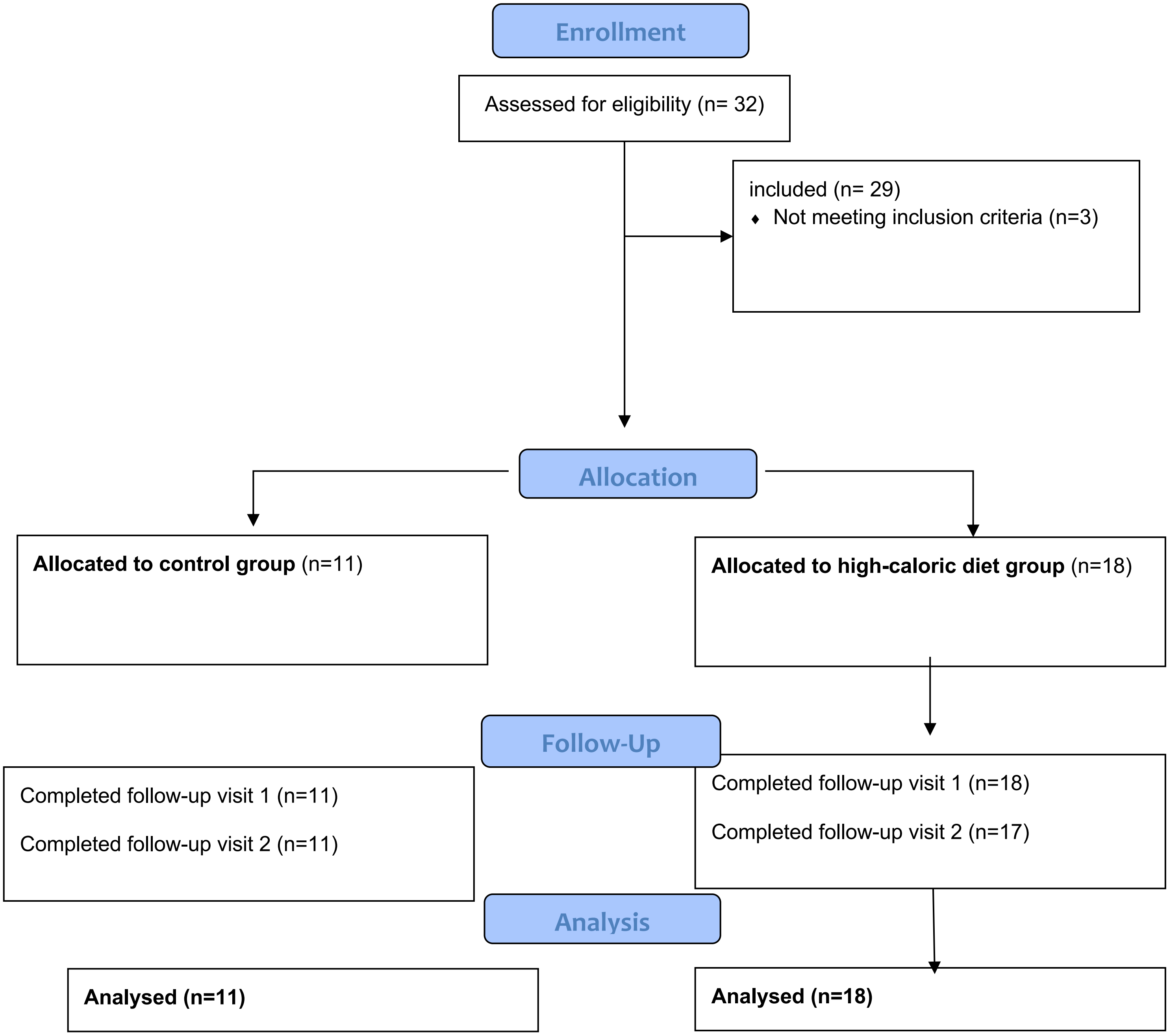

Supplementary Table 1. Nutritional composition of provided snacks

Description (Label in German)	Portion [g]	Energy [kcal]	Fat [%]	Fat [g]	Carb [%]	Carb [g]	Prot [%]	Prot [g]	Fiber [g]	Sug [g]	SFA [g]
Milka Brownies	25	118	28	8	21	12	2	1		10	3
Funny Frisch Chips	50	262	56	17	38	25	5	3	2	1	2
Ültje Erdnüsse	50	307	72	25	9	7	17	13	4	3	5
LU TUC Classic 75 g	75	349	36	14	56	48	7	6	2	5	6
Corny Riegel Schoko	25	113	35	4	59	17	6	2		9	3
Corny Riegel Erdbeer	25	111	34	4	60	16	6	2		6	3
Corny Riegel Schoko	25	110	32	4	63	17	5	1		9	3
Corny Riegel Cocos	25	114	44	6	51	14	5	1		8	5
Kinderschokolade 12,5g	13	70	55	4	39	7	6	1		7	3
Pickup Riegel Leibnitz 28g	28	142	46	7	49	17	6	2		10	4
Kinder Bueno (2 Riegel = 43 g)	43	243	58	16	36	21	6	4		18	7
Kitkat Nestle Mini 16 g	16	84	47	5	46	9	6	1		8	3
Twix (2 Riegel = 50g)	50	246	43	12	53	32	4	2			7
Snickers 50g	50	240	42	11	51	30	7	4		26	
Bounty Riegel 28.5 g	29	138	48	7	49	17	3	1		14	6
Mars Riegel 51g	51	229	33	9	63	36	3	2	1	33	4
M&M Packung 45 g	45	227	44	11	48	27	8	4			5
Ültje Studentenfutter 50g Pack	50	267	60	18	27	18	12	8	2	13	3
Bifi Original 1 Stk	25	126	79	11	1		20	6			5
Bifi Roll	50	235	62	17	25	15	13	8			
Knoppers 1 Stk	25	136	54	8	39	13	7	2		9	5

Ethics application (English translation of German document)

to the study

" Influence of high-calorie food intake on the insulin sensitivity of the human central nervous system "

Version 4.3

Head of the study and contact person
Dr. Stephanie Kullman
Institute for Diabetes Research and Metabolic Diseases (IDM) of the Helmholtz Center Munich at the University of Tübingen
Otfried-Müller-Str. 47
72076 Tübingen
Tel. 07071/2987703; Fax 07071/295706

other participants

Maike Borutta (MTA), Institute for Diabetes Research and Metabolic Diseases (IDM) at the Helmholtz Center Munich at the University of Tübingen

Dr. Ralf Veit, Institute for Diabetes Research and Metabolic Diseases (IDM) of the Helmholtz Center Munich at the University of Tübingen

Prof. Dr. Hubert Preissl
Institute for Diabetes Research and Metabolic Diseases (IDM) of the Helmholtz Center Munich at the University of Tübingen

Investigators

PD Dr. med. Martin Heni
Medical Clinic IV, University of Tübingen
Dr. med. Robert Wagner
Medical Clinic IV, University of Tübingen
Dr. med. Caroline Ketterer
Medical Clinic IV, University of Tübingen
Dr. med. Anja Böhm
Medical Clinic IV, University of Tübingen
Carol Metzger
Medical Clinic IV, University of Tübingen

financing
Funding from the German Center for Diabetes Research (DZD) eV

Declaration of consent from the head of the institute
The signatory has read the test plan and agrees to its contents.

Institute Director ______________________ ______________________
Prof. H.-U. Häring Date

Abbreviations

BMI body mass index
BOLD blood oxygen level dependent
CBF cerebral blood flow
fMRI functional magnetic resonance imaging
CNS central nervous system

1. Background

Insulin action and inflammation in the central nervous system

The uptake of glucose into nerve cells occurs via an insulin-independent mechanism, so that the CNS does not rely on the presence of insulin for glucose metabolism. However, insulin receptors are present in the CNS [1-3] . Little is currently known about the physiological effects of these receptors. Animal experiments have shown an influence on neuronal plasticity [4] . In addition, both intranasal and venous insulin appear to cause changes in eating behavior, emotions, and cognitive functions [5-8] .

Insulin acts as a central appetite suppressant in the brain. Mice with a selective neuronal knockout of the insulin receptor show increased food intake, leading to obesity and insulin resistance [9] . These examples suggest that the cerebral effects of insulin and disorders thereof could be involved in the pathogenesis of numerous diseases, including type 2 diabetes mellitus. “Cerebral insulin resistance” could play a key role in the pathogenesis of type 2 diabetes in particular.

Our own preliminary work using functional magnetic resonance imaging (fMRI) studies shows that intranasal insulin regulates the neuronal function of certain brain regions in a weight-dependent manner and influences peripheral insulin sensitivity [8, 10-14] . In addition to the homeostatic system (the hypothalamus), dopaminergic regions in the brain that are responsible for reward behavior and cognitive control are particularly affected. We find a reduced insulin response in the CNS especially in people with a metabolically unfavorable fat distribution - a lot of visceral fat [8] . Visceral fat produces more inflammatory mediators [15] and increased inflammation levels are in turn closely linked to insulin resistance. Inflammation of the brain (i.e. neuroinflammation) is now also considered a possible cause of central insulin resistance [16, 17] . Gliosis and hypothalamic inflammation are newly discovered mechanisms contributing to obesity/prediabetes [18, 19] .
Interestingly, rodent models following a high-calorie diet show that these inflammatory mechanisms occur rapidly in the brain, even before the animals gain weight [18] . In humans, it has been shown, among other things, that a short-term increase in calories, especially from carbohydrates and fats, reduces insulin sensitivity in the body and increases inflammatory parameters in the blood [20, 21] .
Whether a high-calorie diet can trigger insulin resistance or inflammation in the human **brain** is currently not known. Neuroinflammation can be detected non-invasively in humans. In our current studies, we examine the quantitative water content of the brain using proton density [22, 23] . In an initial evaluation on 60 test subjects, we were able to show that BMI significantly correlates with the water content in the hypothalamus. Whether this is related to a reduced insulin response remains to be shown.

2. Aim of the study

It is not yet known in humans whether acutely induced inflammation, triggered by short-term high-calorie food intake, modulates insulin action in the body, particularly in the brain.
The aim of the study is to investigate the effects of a five-day high-calorie diet in healthy young male subjects on peripheral and brain insulin sensitivity as well as on eating behavior, mood and cognition.

<u>We examine the effects on:</u>

- insulin action in the periphery using an oral glucose tolerance test
- the body fat distribution using MRI
- the intranasal insulin effect in the brain using functional magnetic resonance imaging (fMRI)
- the inflammatory processes in the brain by quantifying water content using proton density and diffusion weighted imaging
- cognition and reward behavior using standardized behavioral tests and questionnaires
- the mood using questionnaires
- eating behavior using questionnaires and a snack test

3. Study population

To record the effect of high-calorie food intake on peripheral and brain insulin action, male participants of normal weight will be recruited.

The test subjects are recruited through circular emails and notices at the University Hospital of Tübingen and the University of Tübingen.

3.1. Inclusion criteria:

- normal glucose tolerance
- Non-smoker
- BMI between 19 and 24 kg/m2
- Age between 18 and 29 years
- Less than 2 hours of physical activity per week
- Understanding of study explanations and instructions
- Consent to information in the event of unexpected, proven pathological findings.

3.2. Exclusion criteria

- Vegan or vegetarian diet as well as lactose and gluten intolerance
- Food allergies (e.g. nut allergies)
- Night shift work
- Competitive athletes
- People who wear non-removable metal parts in or on their body, such as:
  Pacemaker
  artificial heart valves
  Metal prostheses
  implanted magnetic metal parts (screws, plates from operations)
  Metal splinters/shrapnel
  fixed braces
  Retainers go over more than five teeth
  Acupuncture needle
  Insulin pump
  Intraport
- Tattoos, permanent make-up, etc.
  People with limited temperature sensitivity and/or increased sensitivity to heating of the body
- Cardiovascular disease cannot be ruled out, such as manifest coronary heart disease, heart failure greater than NYHA 2, previous heart attack, post-stroke condition
- People with hearing problems or increased sensitivity to loud noises
- People with claustrophobia
- Minors or subjects unable to consent are also excluded
- Subjects who have had an operation less than 3 months ago
- Acute illness or infection within the last 4 weeks
- Neurological and psychiatric diseases
- Taking centrally acting pharmaceuticals

- Subjects with hemoglobin values Hb<14g/dl
- Other illnesses that, in the opinion of an investigator, question the success of the study or indicate a risk to the test subject
- Allergic illnesses to one of the substances used

3.3. Case number estimate
We will recruit 32 normal weight male subjects. Based on our previous experience, we expect a drop-out rate of a maximum of 10%, so we assume at least 29 complete data sets. This number is based on our own previous studies in which we used fMRI to examine insulin effects in the brain in normal and overweight subjects [8, 24] . A large effect size of *Cohen's d* greater than 1 could be achieved with 20 subjects to detect obesity-associated insulin resistance [24] .

4. Study procedure and investigation methods (Figure 1)

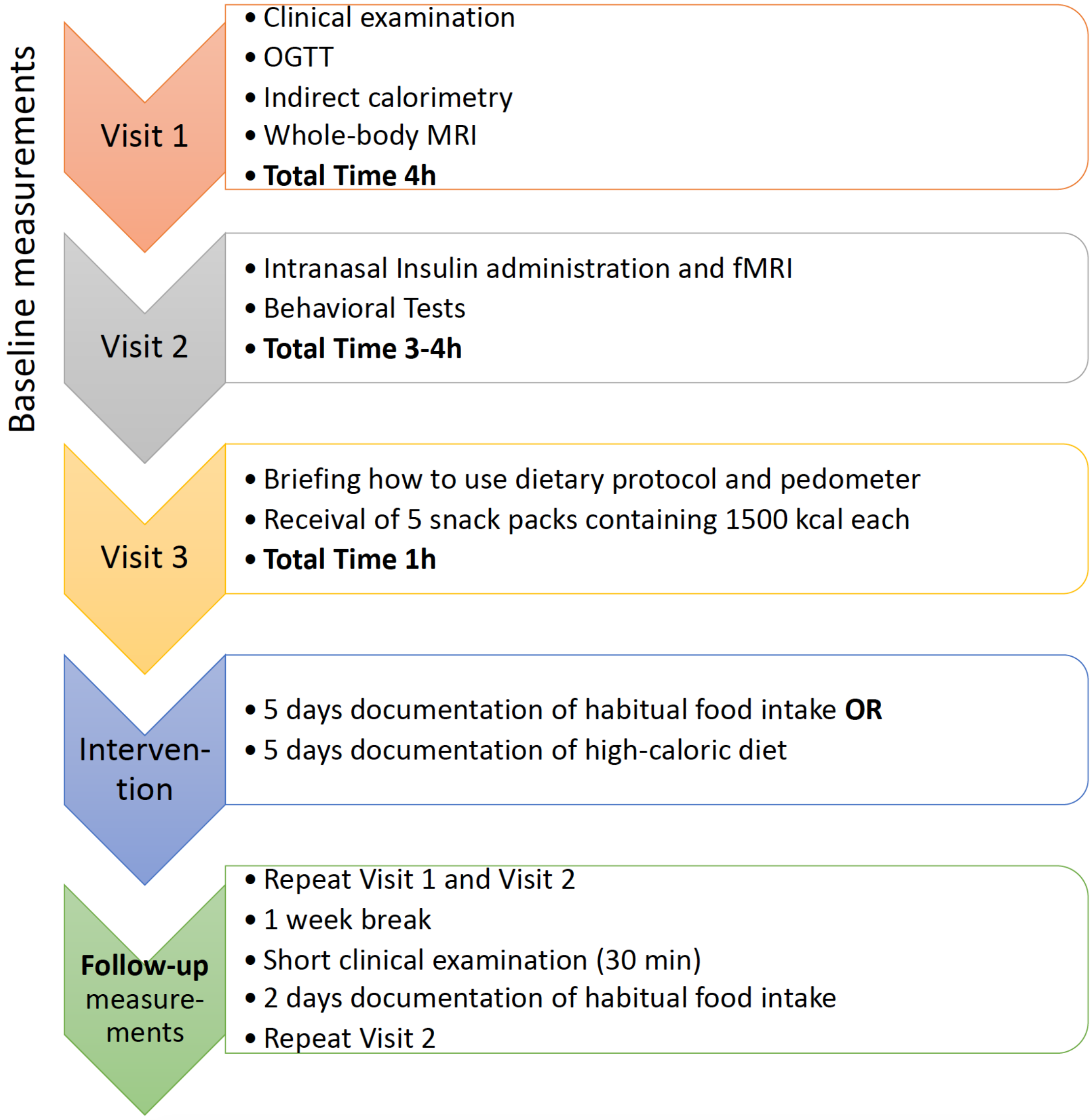

4. Visit 1 to record the peripheral insulin effect and body fat distribution

Before the study begins, participants are informed about the study by one of the experimenters. The subjects will be clinically examined by a doctor beforehand as part of the study, including determination of electrolytes, creatinine, transaminases, coagulation, HbA1c, thyroid hormones and a urine test. Impaired glucose tolerance is ruled out using an oral glucose tolerance test.
In addition, the test subjects are questioned about previous neurological and psychiatric illnesses during a detailed anamnesis. Each test subject will also be tested for dietary habits using a questionnaire (source: aid information service consumer protection , nutrition, agriculture). Mental disorders are recorded using the health questionnaire (PHQ-D) [25] .

4.1. Anthropometric measurement

The test subjects' height and weight as well as waist circumference and hip circumference are documented.
Bioelectrical impedance analysis (BIA) is used to determine body fat percentage. A weak alternating current is used to create an electromagnetic field in the body via two electrodes.

The voltage drop and the phase shift of the signal voltage are measured via two additional electrodes inside this field (four-wire measurement).
In addition, blood pressure and pulse as well as body temperature are measured. An ECG recording is also carried out.

4.2. Indirect calorimetry
by measuring the production of CO_2 and the consumption of O_2 using a transparent hood that is positioned over the head of the lying subject. The measurement takes approx. 30 minutes, is non-invasive and therefore does not place any strain on the test subject. A calorimetry device from Carefusion (Vmax 29) is used .

4.3. Oral glucose tolerance test – OGTT
The examination is carried out after an overnight fasting period. A venous access (Abbocath 20G) is placed in an elbow vein. After a basal blood sample, the test subject drinks 75 g of a standardized glucose solution. The sampling schedule is: 0, 30, 60, 90, 120 min. In addition to glucose, metabolically important factors such as insulin, proinsulin, C-peptide and free fatty acids are determined. In total, around 85 ml of blood is taken for this test.

4.4. Magnetic resonance imaging and spectroscopy (3 T)
This examination allows the precise determination of the amount of body fat compartments and determination of ectopic fat depots, such as liver fat. The examination is carried out in the morning on an empty stomach, before the OGTT. It will last approximately 45-60 minutes. Before the spectroscopic measurements, images are taken using MR imaging (MRI). Volumes of the adipose tissue compartments of total body fat (TAT), visceral adipose tissue (VAT), and abdominal subcutaneous adipose tissue (SCAT) are carefully recorded using axial umbilical T1-weighted MRI. The liver fat content is denoted by “ proton magnetic resonance spectroscopy ”.

5. Visit 2 to record the brain insulin effect and inflammation

The experiment aims to examine insulin effects in the brain using functional MRI. Insulin levels in the central nervous system are increased by intranasal administration. For this purpose, an insulin solution is used in the form of an intranasal spray. The insulin used is human insulin.
The insulin dose to be administered is 160 U/d (4x40 U). This dosage is based on our previous experiments and numerous studies by other research groups with this dosage. An increase in the level in the cerebrospinal fluid can be observed after insulin administration as a nasal spray after just 10 minutes and peaks 30 minutes after administration [26] . The interval between visit 1 should be at least 3 days and no longer than 4 weeks.
This examination is carried out in the morning in a fasted state (last meal at least 12 hours ago) (3T Prisma at the University Hospital of Tübingen or Hochfeld-MRT Center, MPI, Tübingen). On this day, only a blood sample is taken to confirm the subject's fasting state and to rule out acute illnesses (e.g. CRP, depending on clinical indications). There are also behavioral tests following the fMRI measurement.
The exact timing of the measurement can be seen in the following overview, whereby the start is always set at 8 a.m. (on the left in the table: indication of the time duration for each section):

20 min	Welcome, briefing, clarification of questions,<br>Blood sample for glycemia, insulinemia
40 minutes	functional magnetic resonance imaging<br>1) Anatomical image of the CNS<br>2) Quantitative measurement of proton density<br>3) Brain activity at rest<br>4) Single- voxel spectroscopy (measurement of brain metabolites)

	The individual blocks are separated by short breaks
5 mins	Intranasal administration of insulin
20 min	Break outside the scanner<br>Filling out questionnaires (duration 10 min)
40 minutes	functional magnetic resonance imaging<br>5) Brain activity at rest<br>6) Single- voxel spectroscopy<br>7) Brain response (fMRI -BOLD) to visual food stimuli
60 minutes	Memory test and evaluation of previously seen food-related images & further behavioral tests

The total duration of an examination day is around 3.5 hours.

5.1. fMRI measurement (3 Tesla)

The neurophysiological measurements on the magnetic resonance tomograph for the intranasal insulin experiment consist of two parts. The first part takes place basally, with the second being carried out after the intranasal administration of insulin. The break between measurements is used to fill out questionnaires about eating behavior and mood. The fMRI measurements each take approx. 40 minutes, with the subjects having no task during the basal measurement. An ECG is recorded during the MRI measurement.

During the first MRI measurement, an anatomical image of the brain is taken and a measurement is carried out to quantitatively quantify water content [22, 23] . During the first and second fMRI measurements, brain activity is measured at rest. Absolute cerebral blood flow (CBF) enables brain activity to be recorded in physiologically defined and therefore comparable units (ml/100g/min) without stimulation. To capture coherent patterns of spontaneous blood oxygen level dependent (BOLD) fluctuations in the resting state (resting-state) is carried out immediately after the CBF measurements of a standard fMRI BOLD sequence. Furthermore, in the first and second measurements, a spectroscopic examination (^{1}H-MRS) of the gray and white matter is recorded in order to record metabolites that are modulated by inflammatory processes and intranasal insulin. Only in the second fMRI measurement are the subjects asked to look at food pictures and to press a button when a cross appears. This ensures their attention.

The MRI examinations are carried out by people with magnetic resonance imaging certification; at least two people are on site for each measurement. The investigators have extensive experience with the examination methods to be used.

Questionnaires:

Between the fMRI measurements, the subject's subjective state is checked using a visual analogue scale to record needs (eating, drinking, etc.). Mood is recorded by the German form of the positive and negative affect schedule (PANAS) [27] . Other questionnaires on eating behavior include the Eating Behavior Questionnaire [28] and the German version of the Food Cravings Questionaire [29, 30] .

5.2. Behavioral tests:

Memory and attention tests:

After the fMRI experiment, the subjects will take part in behavioral tests after a 15-minute break. During the break, the test subjects are given a glass of water to drink. The subjects are then presented with the food-relevant images from the second fMRI measurement on a laptop.

The subjects are asked to remember whether they have seen the images before or not (40% of the images are unknown) and then to indicate on a scale how certain they are about their decision. Furthermore, the test subjects should rate how much they want to eat the presented food images. Further tests are used to assess the subject's attention (Trail- making Test A&B) and memory (Hopkins-verbal learning test- revised).

Approach and avoidance learning for rewards and punishments
The dopamine system can be studied through the approach and avoidance behavior learning task for rewards and punishments [31, 32] . Here, test subjects have to learn that certain images are more likely to lead to rewards (e.g. winning €1) if one acts, whereas other images are more likely to lead to rewards if one does not act. Similarly, they have to learn which actions after certain pictures are more likely to lead to successfully avoiding punishment (the loss of €1). Using a mathematical model of behavior, the person's sensitivity to rewards and punishments, as well as their learning rate and general tendency to act, can be determined.

Cookie test:
This is a test to examine the consumption of snacks in a casual atmosphere (cookie test). Three plates of cookies are placed on a table, each containing a different type and labeled cookie A, B and C. The three varieties include the classic TUC cookies, rice waffles and chocolate cookies. 15 cookies of each type are broken into bite-sized pieces. This ensures that a significant amount can be eaten without the plates appearing empty and thus the participants do not limit their consumption. A glass of mineral water is also provided. The subject is instructed to rate each type of cookie on a visual analogue scale to assess taste (0-100 for each). The test subjects are asked to rate the cookies as accurately as possible. As many cookies may be eaten as the test subject feels necessary in order to be able to make an accurate assessment of the taste. In addition, after completing the assessment, the test subject is informed that they are welcome to help themselves to the leftover cookies. Consumption is measured by weighing the cookies before and after the test.

5.3. Preparation of infusion solution and dosage

The investigational product is highly concentrated insulin Actrapid ® (100 IU/ml), which is supplied for intranasal administration in spray bottles in which 1 actuation contains 40 IU insulin. The spray bottles are filled according to GMP-compliant procedures in the Tübingen University Pharmacy.

6. Visit 3 - Nutritional advice

A nutritionist from Internal Medicine Department IV will instruct the test subjects on how to keep a food diary and how to use the pedometer. The test subjects receive **five *snack packs***, each containing **1500 kcal** . Beforehand, the test subjects rate the snacks based on their taste. Only products rated as very tasty are selected for the snack pack. When it comes to the daily composition, care is taken to ensure that the nutritional values of the snacks correspond to a high-calorie Western diet (approx. 50% carbohydrates, 35% fat and 15% protein). Subjects are instructed to avoid alcohol. Furthermore, the test subjects are asked to refrain from any sporting activity during the intervention (see point 7).
Example of a snack pack:
- 2*30g peanut pack (372 kcal)
- 2x35g sponge cake (338 kcal)
- 58g Twix (285 kcal)
- 2*25g muesli bars (228 kcal)
- 1*50g chips (265 kcal)

7. Intervention

7.1 Habitual food intake ("normal" diet)
After the consultation, the pedometer is activated and the test subject documents his habitual food intake in a diary for 5 days. From this point on, the subjects are asked to refrain from physical activity and to walk fewer than 4,000 steps. Cycling is also prohibited during the intervention.

7.2 Pedometer
To record daily physical activity, study participants will be given a Fitbit accelerometer Inc issued. This should be worn on the wrist or hip for the entire duration of the study to monitor changes in physical activity. These show the daily recording duration (time accelerometer was worn), the daily movement (everyday movement) and the daily intensive movement (sport). After registering with an email address, the data can be exported as an Excel table. The data processing takes place on a server owned by the manufacturer, which is subject to American data protection regulations. In order to ensure the anonymity of our test subjects, we will create a functional email account for each of the devices currently in use, which will be used to log in to the manufacturer's website (http://www.fitbit.com/de). During the test subjects' regular visits, the data is read out via the assigned email account and stored by us. Only the employees responsible for this know the association between device, email account and test subject. The data is stored on the device in a pseudonymized form according to EOS.

7.3 . High calorie food intake
The test subject is instructed to maintain their habitual diet and additionally consume the high-calorie snacks provided to him (1500 kcal per day). Control subjects are asked to maintain their habitual diet without receiving additional snacks. All meals should be recorded by the test subjects in a diary and photographed with a cell phone camera for later evaluation.
7.3.1. Determination of peripheral and brain insulin effects after a short-term high-calorie diet
On the sixth and seventh days after starting the high-calorie diet, visits 1 and 2 are repeated (see points 4 and 5).

7.4. Clinical examination
On the twelfth to fourteenth day after starting the high-calorie diet, the test subject is again clinically examined by a doctor. This includes recording blood pressure, fasting glucose and insulin, blood lipids, inflammatory markers and hormones, as well as a urine test. The total duration is 30 minutes .

7.5. Habitual food intake after a high-calorie diet ("normal" diet)
From the thirteenth to a maximum of seventeenth day after starting the high-calorie diet, the test subject should document his habitial food intake for two days (see point 7.1).

7.5.1. Determination of the peripheral and brain insulin effect after a "usual " diet
Visits 2 is then repeated (see points 4 and 5). The time of the visits is between the fifteenth and nineteenth day after the start of the high-calorie diet.

8. Blood samples
At visit 1, a total of around 100 ml of blood will be taken. At visit 2, a maximum of 10 ml of blood will be taken. During the clinical examination after the intervention (see point 7.4), 50 ml of blood will be taken. In addition to blood sugar, insulin, C-peptide, free fatty acids and other hormones, routine parameters are also determined in order to rule out an acute illness or similar.
Material for the determination of further metabolites or hormones is frozen for a maximum of 15 years.

9. Compensation

The compensation for this study is €600. This amount seems appropriate, as the subjects take part in 6 appointment visits for around 3-4 hours each, nutritional instructions and another clinical examination (1.5 hours). Total duration around 23 hours.

10. Risks for participants and risk-benefit assessment

a) Functional magnetic resonance tomography is generally considered a low-risk and safe examination method. The examination is painless. Negative effects on the human body are not known at the magnetic field strengths used and are not to be expected based on the current state of science. However, in rare individual cases, complications are possible that may require further treatment: mild to moderate headaches can occur, but these usually go away without treatment. Ringing in the ears usually goes away after the examination, but can also persist. There may be a brief feeling of dizziness (like motion sickness) or sensory irritation when entering the topograph. The irradiated waves lead to heating of the tissue, which is sometimes noticeable, can lead to sweating and can also lead to skin irritation due to make-up containing metal and tattoos. Injuries to the patient's skin, soft tissue and nerves caused by metal objects accidentally placed in the magnetic field have been described. Therefore, no make-up should be worn during fMRI examinations and all metal-containing objects such as keys, coins, belt buckles, watches, small electrical devices, credit cards or clothing with larger metal applications (e.g. bra) should be removed.

b) After intranasal administration of insulin, a temporary feeling of dryness in the nasal mucosa may occur. This does not result in any changes to the mucous membrane and is not dangerous. Otherwise there is no risk with intranasal administration, neither for the respiratory tract nor for the brain. There are no known risks or side effects from measuring brain activity using magnetic resonance imaging.

c) Blood is taken on the examination days by puncturing a vein using a butterfly cannula. On the screening day , the subject's blood will be drawn several times during the oral glucose tolerance test. This is done via a venous access (Abbocath 20G), which is placed in a vein in the back of the hand. The venous punctures can be slightly painful and rarely lead to local irritation, bleeding, bruising and inflammation of the blood vessels as well as incorrect puncture of other vessels and nerves. Thrombosis and localized infections around the needle or cannula insertion sites, as well as infections that spread to the entire body, cannot be completely ruled out.

11. Expected Benefit:
Personal benefit for the test subject: The test subject receives extensive laboratory tests on routine parameters.

General benefit: An increase in knowledge about the central nervous system insulin action in humans and its influence through nutrition is expected. These findings can be used in further studies at other institutions and in our working group to better understand the pathogenesis of obesity and diabetes mellitus and to evaluate therapeutic approaches.

12. Individual termination of studies

The study participants can withdraw from the study at any time and without giving reasons, without incurring any disadvantages. Participation in the study is strictly voluntary.

13. Data Protection

As part of the study " Influence of high-calorie food intake on the insulin sensitivity of the human central nervous system " personal data (name, birthday, address, previous findings, study-related findings including imaging procedures, results of study-related examinations, etc.) are collected and processed.
If necessary, medical data is also included in the processing. The data is documented and archived pseudonymously in a protected electronic database to which only authorized employees, including doctoral students who are obliged to maintain professional and data secrecy, have access. In order to check that the treatment data has been correctly transferred from the medical record to the encrypted study database, authorized persons (so-called monitors) are allowed to view the personal disease data that is related to the study. All employees involved are subject to confidentiality.
The data collected as part of the study can also be used and further processed for future research projects at the clinic or institute.
The pseudonymized data is processed and used on survey forms and electronic data carriers usually for a period of 15 years, provided that the purpose of the study, e.g. B. does not require a longer storage period when entering into a database and in long-term studies.
The information obtained in the course of this study can also be transmitted for scientific purposes to cooperation partners within the scope of the European General Data Protection Regulation and to cooperation partners outside the European Economic Area, i.e. in countries with a lower level of data protection (this also applies to the USA).
The research results from the study will be published anonymously in specialist journals or in scientific databases. When the research results are published, the identities of the study participants will not be revealed. However, the on-site investigators can use a patient list to trace the data back to the person in case of questions.
The test subject can request information about their stored data at any time and has the right to have incorrect data corrected. The study participant can also request at any time that their data be deleted or anonymized so that a connection to the person can no longer be established.
The head of the study (Stephanie Kullmann) is responsible for data processing and compliance with statutory data protection regulations.
Complaints can be made to the data protection officer of the University Hospital of Tübingen or the state data protection officer of the state of Baden-Württemberg.
For the collection, storage, use and disclosure of study participant data, express consent is required by signing the data protection consent declaration.

14. Ethical concerns

Statement on the Declaration of Helsinki and the GCP

This study will be conducted in accordance with the ethical principles of the Declaration of Helsinki (2013 version, Fortaleza) and the International Conference on Harmonization Good Clinical Practice Guideline (ICH-GCP Guideline), as well as applicable legal regulations.

Informing study participants

The test subjects are informed in advance that any unexpected morphological findings or other serious health abnormalities will be reported to them by a doctor. Only subjects who agree to this will be included.
See also appendix: information for test subjects and declaration of consent.

15. Storage of study documents

S. Data protection

17. References:

1. Baskin, DG, et al., *Insulin in the brain.* Annu Rev Physiol, 1987. **49** : p. 335-47.
2. Schwartz, MW, et al., *Insulin in the brain: a hormonal regulator of energy balance.* Endocr Rev, 1992. **13** (3): p. 387-414.
3. Unger, JW, JN Livingston, and AM Moss, *Insulin receptors in the central nervous system: localization, signaling mechanisms and functional aspects.* Prog Neurobiol, 1991. **36** (5): p. 343-62.
4. Huang, CC, CC Lee, and KS Hsu, *The role of insulin receptor signaling in synaptic plasticity and cognitive function.* Chang Gung Med J, 2010. **33** (2): p. 115-25.
5. Kullmann, S., et al., *Brain Insulin Resistance at the Crossroads of Metabolic and Cognitive Disorders in Humans.* Physiol Rev, 2016. **96** (4): p. 1169-209.
6. Hallschmid, M., et al., *Postprandial Administration of Intranasal Insulin Intensifies Satiety and Reduces Intake of Palatable Snacks in Women.* Diabetes, 2012.
7. Benedict, C., et al., *Intranasal insulin to improve memory function in humans.* Neuroendocrinology, 2007. **86** (2): p. 136-42.
8. Kullmann, S., et al., *Selective Insulin Resistance in Homeostatic and Cognitive Control Brain Areas in Overweight and Obese Adults.* Diabetes Care, 2015.
9. Bruning, JC, et al., *Role of brain insulin receptor in control of body weight and reproduction.* Science, 2000. **289** (5487): p. 2122-5.
10. Heni, M., et al., *Nasal insulin changes peripheral insulin sensitivity simultaneously with altered activity in homeostatic and reward-related human brain regions.* Diabetologia, 2012. **55** (6): p. 1773-82.
11. Kullmann, S., et al., *Intranasal insulin modulates intrinsic reward and prefrontal circuitry of the human brain in lean women.* Neuroendocrinology, 2012. **97** (2): p. 176-82.
12. Stingl, KT, et al., *Insulin modulation of magnetoencephalographic resting state dynamics in lean and obese subjects.* Front Syst Neurosci, 2010. **4** : p. 157.
13. Guthoff, M., et al., *Insulin modulates food-related activity in the central nervous system.* J Clin Endocrinol Metab, 2010. **95** (2): p. 748-55.
14. Heni, M., et al., *Central insulin administration improves whole-body insulin sensitivity via hypothalamus and parasympathetic outputs in men.* Diabetes, 2014. **63** (12): p. 4083-8.
15. Fontana, L., et al., *Visceral fat adipokine secretion is associated with systemic inflammation in obese humans.* Diabetes, 2007. **56** (4): p. 1010-3.
16. Thaler, JP, et al., *Obesity is associated with hypothalamic injury in rodents and humans.* J Clin Invest, 2011. **122** (1): p. 153-62.
17. Jastroch, M., et al., *The hypothalamic neural-glial network and the metabolic syndrome.* Best Pract Res Clin Endocrinol Metab, 2014. **28** (5): p. 661-71.
18. Dorfman, MD and JP Thaler, *Hypothalamic inflammation and gliosis in obesity.* Curr Opin Endocrinol Diabetes Obes, 2015.
19. Garcia-Caceres, C., CX Yi, and MH Tschop, *Hypothalamic astrocytes in obesity.* Endocrinol Metab Clin North Am, 2013. **42** (1): p. 57-66.
20. Tam, CS, et al., *Short-term overfeeding may induce peripheral insulin resistance without aging subcutaneous adipose tissue macrophages in humans.* Diabetes, 2010. **59** (9): p. 2164-70.
21. Knudsen, SH, et al., *Changes in insulin sensitivity precede changes in body composition during 14 days of step reduction combined with overfeeding in healthy young men.* J Appl Physiol (1985), 2012. **113** (1): p. 7-15.
22. Abbas, Z., et al., *Quantitative water content mapping at clinically relevant field strengths: a comparative study at 1.5 T and 3 T.* Neuroimage, 2015. **106** : p. 404-13.
23. Abbas, Z., et al., *Analysis of proton-density bias corrections based on T1 measurement for robust quantification of water content in the brain at 3 Tesla.* Magn Reson Med, 2014. **72** (6): p. 1735-45.
24. Kullmann, S., et al., *Intranasal insulin modulates intrinsic reward and prefrontal circuitry of the human brain in lean women.* Neuroendocrinology, 2013. **97** (2): p. 176-82.
25. Löwe, B., et al., *PHQ-D. Health questionnaire for patients. Karlsruhe, Germany:Pfitzer.* 2002.
26. Born, J., et al., *Sniffing neuropeptides: a transnasal approach to the human brain.* Nat Neurosci, 2002. **5** (6): p. 514-6.

27. Krohne, HW, et al., *Examination of the German form of the Positive and Negative Affect Schedule (PANAS).* Diagnostica, 1996. **42** : p. 139-156.
28. Pudel, D. and J. Westenhöfer, *Eating Behavior Questionnaire (FEV). Hand instruction. Göttingen:Hogrefe.* 1989.
29. Meule, A., T. Hermann, and A. Kubler, *A short version of the Food Cravings Questionnaire-Trait: the FCQ-T-reduced.* Front Psychol, 2014. **5** : p. 190.
30. Meule, A., et al., *On the differentiation between trait and state food craving: Half-year retest-reliability of the Food Cravings Questionnaire-Trait-reduced (FCQ-Tr) and the Food Cravings Questionnaire-State (FCQ -S).* J Eat Disord, 2014. **2** (1): p. 25.
31. Guitart-Masip, M., et al., *Differential, but not antagonist, effects of L -DOPA and citalopram on action learning with reward and punishment.* Psychopharmacology (Berl), 2014. **231** (5): p. 955-66.
32. Guitart-Masip, M., et al., *Go and no-go learning in reward and punishment: interactions between affect and effect.* Neuroimage, 2012. **62** (1): p. 154-66.
