# Supplementary material for: A short-term, high-caloric diet has prolonged effects on brain insulin action in men
Source: Nat Metab. 2025 Feb 21;7(3):469–77. doi: 10.1038/s42255-025-01226-9 (PMC11946887; doi:10.1038/s42255-025-01226-9)
Supplement: Supplementary file 2 — Reporting Summary [file 42255_2025_1226_MOESM2_ESM.pdf]

nature portfolio

Corresponding author(s): Stephanie Kullmann

Last updated by author(s): Jan 13, 2025

Reporting Summary

Nature Portfolio wishes to improve the reproducibility of the work that we publish. This form provides structure for consistency and transparency in reporting. For further information on Nature Portfolio policies, see our Editorial Policies and the Editorial Policy Checklist.

Statistics

For all statistical analyses, confirm that the following items are present in the figure legend, table legend, main text, or Methods section.

n/a	Confirmed	
☐	☒	The exact sample size (*n*) for each experimental group/condition, given as a discrete number and unit of measurement
☐	☒	A statement on whether measurements were taken from distinct samples or whether the same sample was measured repeatedly
☐	☒	The statistical test(s) used AND whether they are one- or two-sided<br>*Only common tests should be described solely by name; describe more complex techniques in the Methods section.*
☐	☒	A description of all covariates tested
☐	☒	A description of any assumptions or corrections, such as tests of normality and adjustment for multiple comparisons
☐	☒	A full description of the statistical parameters including central tendency (e.g. means) or other basic estimates (e.g. regression coefficient) AND variation (e.g. standard deviation) or associated estimates of uncertainty (e.g. confidence intervals)
☐	☒	For null hypothesis testing, the test statistic (e.g. *F*, *t*, *r*) with confidence intervals, effect sizes, degrees of freedom and *P* value noted<br>*Give P values as exact values whenever suitable.*
☒	☐	For Bayesian analysis, information on the choice of priors and Markov chain Monte Carlo settings
☒	☐	For hierarchical and complex designs, identification of the appropriate level for tests and full reporting of outcomes
☒	☐	Estimates of effect sizes (e.g. Cohen's *d*, Pearson's *r*), indicating how they were calculated

Our web collection on statistics for biologists contains articles on many of the points above.

Software and code

Policy information about availability of computer code

Data collection	No software was used for data collection.
Data analysis	IBM SPSS 27 Statistics; ASLtbx with SPM12 (Wellcome Trust Centre for Neuroimaging; MATLAB v2016a; Software Library FSL version 6.0; Program R version 4.3.3.

For manuscripts utilizing custom algorithms or software that are central to the research but not yet described in published literature, software must be made available to editors and reviewers. We strongly encourage code deposition in a community repository (e.g. GitHub). See the Nature Portfolio guidelines for submitting code & software for further information.

Data

Policy information about availability of data

All manuscripts must include a data availability statement. This statement should provide the following information, where applicable:

- Accession codes, unique identifiers, or web links for publicly available datasets
- A description of any restrictions on data availability
- For clinical datasets or third party data, please ensure that the statement adheres to our policy

Source data related to the tables, figures and extended data figures and tables are provided in this paper. Statistical maps of the brain effects are uploaded to neurovault (https://neurovault.org/collections/MYQHVMQE/).

Research involving human participants, their data, or biological material

Policy information about studies with human participants or human data. See also policy information about sex, gender (identity/presentation), and sexual orientation and race, ethnicity and racism.

Reporting on sex and gender	Previous experimental findings indicate sex differences in the response to brain insulin action affecting appetite, metabolism, and memory function. While increasing circulating estradiol and decreasing testosterone concentrations in healthy young men does not interfere with insulin's effect on food intake, the centrally mediated effect of insulin on peripheral insulin sensitivity in females critically depends on their menstrual cycle phase. Since this is the first study investigating the temporal dynamics of brain insulin action in response to an unhealthy diet, we only evaluated brain insulin action in response to overeating in normal-weight male participants. Whether the findings can be extended to females needs to be investigated in future studies.
Reporting on race, ethnicity, or other socially relevant groupings	Participants with high levels of education and a high socio-economic status are enriched in this study population. Given the repeated measurement design and the incusion of a control group, we do not expect an impact on the results.
Population characteristics	29 male participants were aged 19 to 27 years, normal-weight (BMI range 19 – 25 kg/m2), nonsmokers, weight-stable for at least 3 months before the study visits, nondieters, no vegan or vegetarian dieters, no food allergies, exercising less than 2h a week, not working at night, not taking medication, and with no history of diabetes, eating disorders, illicit drug use, or other medical diagnoses. Gender was determined based on self-report and supported by testosterone measurements.
Recruitment	Participants were recruited by University-wide email anouncements. Participants were not aware at the start of the study, if they were assigned to HCD or control group. As only young students were recuited, there was a selection bias towards high level of education but low income. This could be a selection bias. We do not expect this to impact our results, as both control and HCD group did not differ in this regard. Furthermore, the repeated measurement design minimize the impact of this bias.
Ethics oversight	Participants provided written informed consent in compliance with the University of Tübingen ethical committee. The study (813/2017BO2) received approval by the local ethics committee in January 2018 (Ethics Committee of the Medical Faculty of the Eberhard Karls University and the University Hospital Tübingen) and was conducted according to the relevant guidelines and regulations. The study is basic experimental study involving humans (BESH) and was registered at ClinicalTrials.gov (NCT03590561). A compensation of €600 was provided after completion of the study (total of 23 hours over 6 visits).

Note that full information on the approval of the study protocol must also be provided in the manuscript.

Field-specific reporting

Please select the one below that is the best fit for your research. If you are not sure, read the appropriate sections before making your selection.

☒ Life sciences ☐ Behavioural & social sciences ☐ Ecological, evolutionary & environmental sciences

For a reference copy of the document with all sections, see nature.com/documents/nr-reporting-summary-flat.pdf

Life sciences study design

All studies must disclose on these points even when the disclosure is negative.

Sample size	A previous study investigating brain insulin action (Kullmann et al 2015 Diab Care), showed a large effect size of Cohen's d greater than 1 could be achieved with 18 subjects per group to detect obesity-associated insulin resistance. Assuming a 2-sided t-test of, α=0.05 and 80% power, a total sample size of at least 25 persons was calculated. To compensate for potential drop outs, n=7 were additionally recruited.
Data exclusions	We miss one fMRI measurement timepoint of the second follow up from N=1. This is presented in the supplementary file (consort file)
Replication	We confirm that the regions identified to be responsive to intranasal insulin in the current study we found in previous fMRI studies (by our and other groups) Ref: Kullmann et al Lancet D&E 2020; Nijssen et al 2023 Neuroendocrinology)
Randomization	The allocation of the groups was not randomized as the exerimental group received instructions by a nutritional scientist how to increase their caloric content, while the control group received instructions how to maintain their regular diet.
Blinding	Persons performing fMRI data acqusition and fMRI data analysis were blinded to experimental groups.

Reporting for specific materials, systems and methods

We require information from authors about some types of materials, experimental systems and methods used in many studies. Here, indicate whether each material, system or method listed is relevant to your study. If you are not sure if a list item applies to your research, read the appropriate section before selecting a response.

Materials & experimental systems

n/a	Involved in the study
☒	☐ Antibodies
☒	☐ Eukaryotic cell lines
☒	☐ Palaeontology and archaeology
☒	☐ Animals and other organisms
☐	☒ Clinical data
☒	☐ Dual use research of concern
☒	☐ Plants

Methods

n/a	Involved in the study
☐	☐ ChIP-seq
☐	☐ Flow cytometry
☐	☒ MRI-based neuroimaging

Clinical data

Policy information about clinical studies
All manuscripts should comply with the ICMJE guidelines for publication of clinical research and a completed CONSORT checklist must be included with all submissions.

Clinical trial registration	NCT03590561
Study protocol	We enclosed the study protocol (English translation) as part of supplement.
Data collection	Data were collected at the University Hospital of Tübingen between June 2018 to March 2020.
Outcomes	From before to after changing the diet compared to a control group, primary and secondary outcomes were the change in brain activity and brain structure (assessed by MRI), change in peripheral insulin sensitivity assessed by five-point 75 gram oral glucose tolerance tests, change in body fat distribution/ intrahepatic fat content by MRI and MRS, and change in reward learning by go/nogo learning task.

Plants

Seed stocks	*Report on the source of all seed stocks or other plant material used. If applicable, state the seed stock centre and catalogue number. If plant specimens were collected from the field, describe the collection location, date and sampling procedures.*
Novel plant genotypes	*Describe the methods by which all novel plant genotypes were produced. This includes those generated by transgenic approaches, gene editing, chemical/radiation-based mutagenesis and hybridization. For transgenic lines, describe the transformation method, the number of independent lines analyzed and the generation upon which experiments were performed. For gene-edited lines, describe the editor used, the endogenous sequence targeted for editing, the targeting guide RNA sequence (if applicable) and how the editor was applied.*
Authentication	*Describe any authentication procedures for each seed stock used or novel genotype generated. Describe any experiments used to assess the effect of a mutation and, where applicable, how potential secondary effects (e.g. second site T-DNA insertions, mosiacism, off-target gene editing) were examined.*

ChIP-seq

Data deposition

☐ Confirm that both raw and final processed data have been deposited in a public database such as GEO.

☐ Confirm that you have deposited or provided access to graph files (e.g. BED files) for the called peaks.

Data access links<br>*May remain private before publication.*	*For "Initial submission" or "Revised version" documents, provide reviewer access links. For your "Final submission" document, provide a link to the deposited data.*
Files in database submission	*Provide a list of all files available in the database submission.*
Genome browser session<br>(e.g. UCSC)	*Provide a link to an anonymized genome browser session for "Initial submission" and "Revised version" documents only, to enable peer review. Write "no longer applicable" for "Final submission" documents.*

Methodology

Replicates	*Describe the experimental replicates, specifying number, type and replicate agreement.*
Sequencing depth	*Describe the sequencing depth for each experiment, providing the total number of reads, uniquely mapped reads, length of reads and whether they were paired- or single-end.*
Antibodies	*Describe the antibodies used for the ChIP-seq experiments; as applicable, provide supplier name, catalog number, clone name, and lot number.*

Peak calling parameters	*Specify the command line program and parameters used for read mapping and peak calling, including the ChIP, control and index files used.*
Data quality	*Describe the methods used to ensure data quality in full detail, including how many peaks are at FDR 5% and above 5-fold enrichment.*
Software	*Describe the software used to collect and analyze the ChIP-seq data. For custom code that has been deposited into a community repository, provide accession details.*

Flow Cytometry

Plots

Confirm that:

☐ The axis labels state the marker and fluorochrome used (e.g. CD4-FITC).

☐ The axis scales are clearly visible. Include numbers along axes only for bottom left plot of group (a 'group' is an analysis of identical markers).

☐ All plots are contour plots with outliers or pseudocolor plots.

☐ A numerical value for number of cells or percentage (with statistics) is provided.

Methodology

Sample preparation	*Describe the sample preparation, detailing the biological source of the cells and any tissue processing steps used.*
Instrument	*Identify the instrument used for data collection, specifying make and model number.*
Software	*Describe the software used to collect and analyze the flow cytometry data. For custom code that has been deposited into a community repository, provide accession details.*
Cell population abundance	*Describe the abundance of the relevant cell populations within post-sort fractions, providing details on the purity of the samples and how it was determined.*
Gating strategy	*Describe the gating strategy used for all relevant experiments, specifying the preliminary FSC/SSC gates of the starting cell population, indicating where boundaries between "positive" and "negative" staining cell populations are defined.*

☐ Tick this box to confirm that a figure exemplifying the gating strategy is provided in the Supplementary Information.

Magnetic resonance imaging

Experimental design

Design type	Resting state
Design specifications	Resting-state cerebral blood flow measurements before and 30 min after intranasal insulin.
Behavioral performance measures	During MRI no behavior was assessed.

Acquisition

Imaging type(s)	Functional and Diffusion
Field strength	3 Tesla
Sequence & imaging parameters	Arterial spin labeling (PASL) images were obtained with a PICORE-Q2TIPS (proximal inversion with control for off-resonance effects (quantitative imaging of perfusion by using a single subtraction) sequence by using a frequency offset corrected inversion pulse and echo planar imaging readout for acquisition. In addition, high-resolution T1-weighted anatomical images were obtained. Diffusion-weighted images (DWIs) were acquired using a echo planar imaging sequence with 35 directions and GRAPPA acceleration factor 2.
Area of acquisition	Whole-brain

Diffusion MRI ☒ Used ☐ Not used

Parameters	An echo planar imaging sequence (70 axial slices, FOV = 220 mm2, slice thickness = 2mm, TE = 54 ms, TR = 6500 ms) with 35 directions (b = 1000 s/mm2) and GRAPPA acceleration factor 2 was acquired. Moreover, eleven interspersed nondiffusion weighted volumes (b0) were recorded. To improve SNR two averages were performed.

Preprocessing

Preprocessing software	Image preprocessing was performed using the ASLtbx with SPM12 (Wellcome Trust Centre for Neuroimaging). Functional images were motion corrected, coregistered to the individual anatomical image, and smoothed (full width half maximum: 6 mm). For Diffusion MRI, standard preprocessing and statistical analyses were performed using FMRIB (Functional Magnetic Resonance Imaging of the Brains's, Oxford University, UK) Software Library (FSL version 6.0).
Normalization	The high resolution T1-weighted image was normalized in Montreal Neurological Institute space (1 x 1 x 1 mm) using SPM12's unified segmentation normalization, and the resulting parameter file was used with the individual coregistered CBF maps in normalized space (3 x 3 x 3 mm).
Normalization template	normalized in Montreal Neurological Institute (MNI305)
Noise and artifact removal	Six head motion parameters. No participant had head motion with more than 2.0 mm maximum displacement or 2.0° of any angular motion.
Volume censoring	NA

Statistical modeling & inference

Model type and settings	For functional MRI, perfusion images were generated by calculating the control-tag differences by using surround subtraction. For accurate CBF quantification (ml x 100g-1 x min-1), we used unique M0 value extracted from a ROI in the cerebro spinal fluid. We used the general kinetic model for absolute perfusion quantification. The absolute change of CBF of each participant from before to after intranasal insulin spray application were used for further statistics (ΔCBF = CBF 30 min post nasal spray minus CBF before nasal spray).
Effect(s) tested	Flexible factorial design (ti investigate group differences, while taking into account within subject variability

Specify type of analysis: ☒ Whole brain ☐ ROI-based ☐ Both

Statistic type for inference (See Eklund et al. 2016)	Whole-brain analyses were performed using a voxel-wise approach in SPM12
Correction	For functional data: A statistical threshold of $p<0.001$ uncorrected and a $p<0.05$ family wise error (FWE) corrected for multiple comparisons was applied on a whole brain level.<br>For tract-based spatial statistics, we used the module randomise in FSL. It is a non-parametric permutation test for inference on statistical maps. For detecting significant clusters corrected for multiple comparisons, we selected the TFCE (Threshold-Free Cluster Enhancement) method optimized for TBSS analyses. Statistical maps were thresholded at $p< 0.05$ (TFCE corrected). The number or permutations was set to 5000.

Models & analysis

n/a	Involved in the study
☒	☐ Functional and/or effective connectivity
☒	☐ Graph analysis
☒	☐ Multivariate modeling or predictive analysis
